# Coyote Range Expansion in the Human‐Modified Tropics of Mesoamerica

**DOI:** 10.1002/ece3.73184

**Published:** 2026-03-02

**Authors:** César R. Rodríguez‐Luna, Fernando M. Contreras‐Moreno, Morelia Camacho‐Cervantes, Daniel Jesús‐Espinosa, Luis A. Trujillo‐Sosa, Alma C. Escobar‐Cifuentes, Alejandro Marmol, Rony García‐Anleu, Martha P. Ibarra‐López, Román Espinal‐Palomino, Anuar D. Hernández‐SaintMartín, Patricio Canul‐Chuc, Víctor Castelazo‐Calva, Marcos Corado, Alberto González‐Gallina, Pedro E. Nahuat‐Cervera, Mircea G. Hidalgo‐Mihart, Carlos N. Ibarra‐Cerdeña

**Affiliations:** ^1^ Departamento de Ecología Humana Centro de Investigación y de Estudios Avanzados (Cinvestav) Unidad Mérida Mérida Yucatán México; ^2^ World Wildlife Fund Inc.‐México Campeche México; ^3^ Instituto de Ciencias del Mar y Limnología Universidad Nacional Autónoma de México, Ciudad Universitaria Ciudad de México México; ^4^ Grupo de Monitoreo Socioambiental Balancán Tabasco México; ^5^ Fundación Defensores de la Naturaleza Ciudad de Guatemala Guatemala; ^6^ Wildlife Conservation Society Guatemala Flores Guatemala; ^7^ Departamento de Ecología y Recursos Naturales Centro Universitario de la Costa Sur, Universidad de Guadalajara Autlán Jalisco México; ^8^ Pronatura Península de Yucatán AC (PPY) Mérida Yucatán México; ^9^ Programme for Belize Guardaparques de la Reserva Río Bravo Orange Walk Belize; ^10^ Unidad de Servicios Profesionales Altamente Especializados Instituto de Ecología A.C. Xalapa, Veracruz México; ^11^ Ekuneil Península de Yucatán Mérida Yucatán México; ^12^ División Académica de Ciencias Biológicas Universidad Juárez Autónoma de Tabasco Villahermosa Tabasco México

**Keywords:** camera‐traps, canid, colonization, dispersal, Mesoamerica, new records

## Abstract

Understanding species range dynamics is central to ecology and biogeography, particularly as global environmental change accelerates range shifts, expansions, and biological invasions. Carnivores are notable for their capacity to exploit human‐modified landscapes, yet most research has focused on temperate regions where apex predator extirpation often facilitates expansion. By contrast, carnivore range expansions in tropical landscapes remain poorly documented. Here we assess the recent southward expansion of coyotes ( *Canis latrans* ) into the human‐modified tropics of Mesoamerica, where they now overlap with apex predator assemblages. We compiled 278 coyote records from areas lacking previous evidence of their presence by integrating data from 44 camera‐trap surveys (1125 cameras deployed, totaling 203,682 camera‐trap/days between 2012 and 2025) and citizen‐science platforms (iNaturalist and the Global Biodiversity Information Facility). To investigate drivers of coyote occurrence, we fitted a generalized linear model (GLM) using the Global Human Modification Index (GHMI), the Normalized Difference Vegetation Index (NDVI), and spatiotemporal factors as predictors. Model results indicated higher probabilities of coyote occurrence in more human‐modified landscapes (higher GHMI) and areas with lower vegetation greenness (lower NDVI). Furthermore, a significant positive temporal trend indicated that the probability of occurrence increased annually. This case highlights how land‐use change facilitates range expansions at continental margins and underscores the conceptual blurring between native range expansion and invasion processes. The ability of coyotes to thrive in both natural and human‐dominated environments suggests continued spatial expansion, reinforcing the need for proactive management strategies grounded in both ecological science and local sociocultural contexts.

## Graphical abstract

Coyotes have expanded their range into southeastern Mexico, Guatemala, and Belize, increasingly occupying human‐modified landscapes. This expansion is ongoing, with rising detection rates over time and records in both disturbed and forested environments, highlighting a paradox where anthropogenic change enables native carnivore expansion.

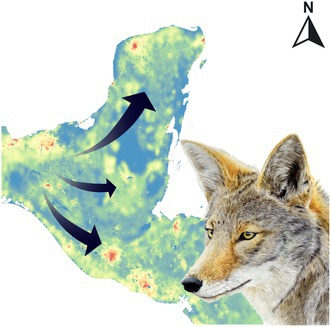

## 1 Introduction

Understanding and predicting how species expand their ranges is central to ecology, including both invasive species establishing in new areas and native species shifting their distributions in response to anthropogenic change. Species ranges are shaped by a combination of ecological tolerances, dispersal capacities, and interactions with increasingly human‐modified environments (Soberón 2007; Pacifici et al. 2017). In this context, large‐scale anthropogenic land‐use change has emerged as a key factor influencing species distributions globally, altering community structure, ecosystem function, and species coexistence (Fischer et al. 2006; Storch et al. 2021). Indeed, a recent assessment on invasive alien species underscores that human‐driven environmental change is a key driver of species redistributions globally, whether involving non‐native species or native range expansions (Roy et al. 2024).

The expansion of adaptable species into humanized landscapes reflects a paradox: while anthropogenic transformation typically degrades habitats and reduces biodiversity, it can also create favorable conditions for generalists and disturbance‐tolerant species. Such environments—characterized by edge effects, fragmented habitats, reduced apex predator pressure, and predictable human‐subsidized resources—often facilitate the success of adaptable carnivores (Gehrt et al. 2009; Bateman and Fleming 2012). Similar patterns of expansion into disturbed environments have been documented for other taxa; for example, invasive freshwater fishes possess traits that confer them high adaptability to novel conditions (Gómez‐Maldonado et al. 2023). In mammals, carnivores (Carnivora) exemplify this dynamic, as their dispersal capacity, ecological flexibility, and interactions with human‐altered landscapes have enabled notable range expansions worldwide (Kelly et al. 2014; Newsome et al. 2015).

The coyote ( *Canis latrans* ) is an archetype of this phenomenon, having undergone one of the most extensive range expansions of any North American mammal (Hody and Kays 2018), a process linked to land‐use change, reduced competition with apex predators, and its behavioral and dietary plasticity (Gompper 2002; Kays et al. 2009). The coyote is a medium‐sized, highly adaptable carnivore that thrives in a variety of habitats, ranging from natural ecosystems to human‐modified environments (Bekoff 1977; Castelló 2018). Its ecological versatility has allowed it to inhabit grasslands, deserts, temperate and tropical forests, as well as peri‐urban and urban areas (Bekoff 1977; Gompper 2002; Gese and Bekoff 2004; Grubbs and Krausman 2009; Servín et al. 2014; Gordillo‐Chávez et al. 2024; Curtis et al. 2025).

Most research on coyote range dynamics has focused on its expansion northward and eastward in temperate regions (Kays et al. 2009; Hody and Kays 2018). By contrast, the species' ongoing southward expansion into tropical Mesoamerica remains less well documented, despite increasing records from human‐modified landscapes in southern Mexico and Central America (Hidalgo‐Mihart et al. 2022; Monroy‐Vilchis et al. 2020). These landscapes, shaped by both historical and recent anthropogenic pressures, offer a complex mosaic of ecological opportunities and constraints (Balée and Erickson 2006; Rivera‐Núñez et al. 2025).

In Mexico, the coyote southward expansion has been documented in southeastern states, including the Lacandon Rainforest and the Maya Forest, but published records remain sparse and geographically fragmented, with reports primarily from peripheral areas such as the northern Yucatán coast and the Calakmul Biosphere Reserve (Sosa‐Escalante et al. 1997; Hidalgo‐Mihart et al. 2013, 2022; Peña‐Mondragón et al. 2014; Contreras‐Moreno et al. 2020). Beyond southern Mexico, multiple records indicate that coyotes have extended their range throughout Central America. Historical records are well established for Costa Rica and Panama (Méndez et al. 1981; Vaughan 1983; Méndez‐Carbajal and Moreno 2014; Cove et al. 2012), while more recent—though still relatively limited—observations further support ongoing geographic expansion into novel environments within these nations (Hody et al. 2019; Carazo‐Salazar et al. 2020; Azofeifa‐Romero et al. 2024). In contrast, records from Nicaragua, Honduras, and El Salvador remain scarce in both the published literature (Baltensperger and Brown 2015; Elvir‐Valle et al. 2019) and open‐access biodiversity databases (e.g., GBIF, iNaturalist), likely reflecting uneven sampling effort rather than true absence. Within this broader regional context, isolated records suggest that coyotes have also reached the Maya Forest of Guatemala and Belize (Platt et al. 1998; Hidalgo‐Mihart et al. 2004; Jones et al. 2020). While habitat modification and land‐use change likely facilitate this expansion, the coexistence of coyotes with apex predators such as jaguars ( *Panthera onca* ) and mountain lions ( *Puma concolor* ) creates a unique ecological context compared to regions where apex predators are absent (Chávez et al. 2007; Ceballos et al. 2021).

In this study, we provide new empirical evidence on the spatial and temporal dynamics of coyote occurrence across underreported regions of southern Mexico, Guatemala, and Belize. We integrate camera‐trap monitoring data and online biodiversity records to map recent occurrences and assess their association with vegetation cover and human land‐use intensity. We further test for temporal trends in detection rates to infer colonization dynamics across the region, offering insights into the ecological and anthropogenic factors facilitating its expansion at the southeastern edge of its continental range. Our findings contribute to broader debates on how human activities reshape species distributions (Jensen et al. 2025), provide a rare case study from a tropical region where carnivore range dynamics remain understudied (Pacifici et al. 2017), and highlight the importance of integrating ecological and social dimensions for anticipating future human‐wildlife interactions.

## 2 Materials and Methods

### 2.1 Study Area

**FIGURE 1:**
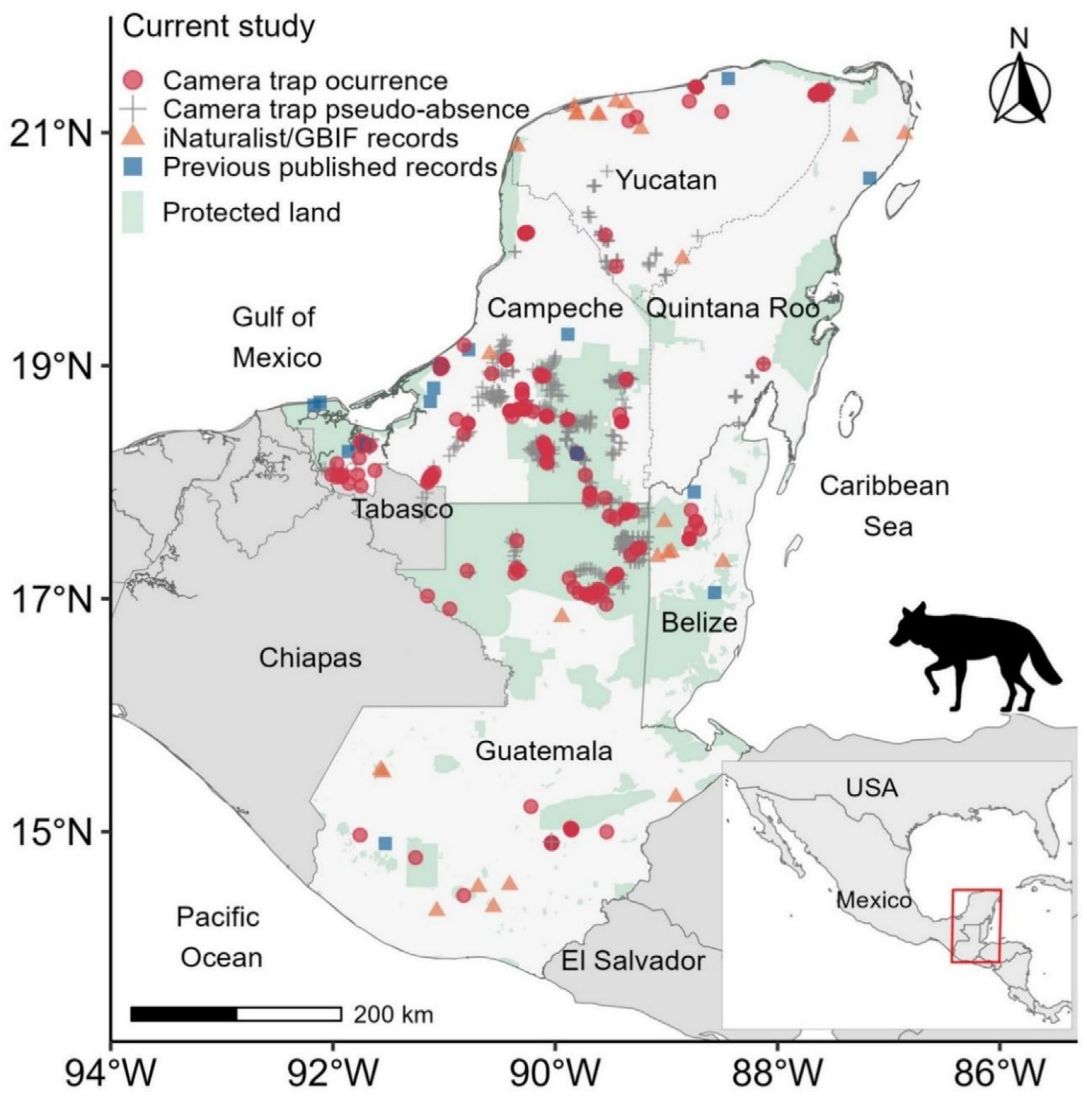
Study area and occurrence records of coyote ( *Canis latrans* ) across southern Mexico, Guatemala, and Belize, classified by data source and acquisition method. Shown are camera‐trap detections, camera‐trap pseudo‐absence locations, records from citizen‐science platforms (iNaturalist and GBIF), and previously published records. Protected areas are indicated for reference. Solid lines represent national borders, while dashed lines indicate state boundaries.

We compiled information on coyote location records from areas where their presence had not been previously documented, focusing on the Yucatán Peninsula in southeastern Mexico, as well as Guatemala and Belize (Figure 1). From a biogeographic perspective, the study region largely corresponds to the Yucatán Peninsula Biotic Province; the drier, northernmost portion of this area is known as the Yucatán Province, while the wetter, southernmost portion is referred to as the Petén Province (Morrone 2005, 2014).

The region is characterized by a pronounced climatic gradient, with lower precipitation levels in the northwest and increasing rainfall toward the southeast, where warm subhumid climates (Aw) predominate (Hodell et al. 2005). In the Yucatán Peninsula, annual average temperatures range from 25.5°C–26.75°C (Torrescano‐Valle and Folan 2015). The rainy season occurs from late May to October, followed by the dry season from November to April (Hodell et al. 2005). Central America and southern Mexico exhibit considerable geomorphological and geographical variability, including complex coastlines, coastal plains, high mountain ranges, interior basins, and expansive plateaus (Pérez et al. 2011). The primary vegetation types include floodplain grasslands and mangroves (wetlands) as well as five main types of forests: low deciduous forests, medium subdeciduous forests, medium semievergreen forests, high evergreen forests, and low flooded forests (Carnevali et al. 2003).

### 2.2 Coyote Location Records

We obtained georeferenced coyote records from 44 camera trap monitoring projects conducted across different parts of the study region between April 2012 and January 2025. Twenty‐nine of these monitoring efforts were carried out in the Yucatán Peninsula (Campeche = 20, Yucatán = 8; Quintana Roo = 1), 10 in Guatemala (El Petén = 7, Zacapa = 2, El Progreso = 1), and five in the Orange Walk District of Belize. Together, these monitoring projects accumulated a total of 203,682 camera‐trap days over a 13‐year period (2012–2025; Table 1).

**TABLE 1:** Camera‐trap surveys conducted to monitor medium and large vertebrates in southern Mexico, Guatemala, and Belize (2012–2025), including survey locations, sampling periods, and sampling effort per survey.

Survey	Country	State/Department	Region	Sampling period	Sampling effort (trap/day)
1	Mexico	Campeche	Champotón	Mar–Nov 2014	13,552
2	Mexico	Campeche	Laguna de Términos	Apr–Nov 2016	5929
3	Mexico	Campeche	Laguna de Términos	May–Oct 2017	11,248
4	Mexico	Campeche	Escárcega‐Calakmul (Balam Ku BR)	Aug–Oct 2018	6398
5	Mexico	Campeche	Calakmul	Jan–Dec 2019	3480
6	Mexico	Campeche	Carmen‐Palizada	Feb–May 2019	2473
7	Mexico	Campeche	Champotón	Feb–Jul 2019	9361
8	Mexico	Campeche	Escárcega‐Calakmul (Balam Ku BR)	Mar–Jun 2019	2748
9	Mexico	Campeche	Escárcega‐Calakmul	Jun–Oct 2019	3480
10	Mexico	Campeche	Carmen‐Palizada	Feb–May 2020	3236
11	Mexico	Campeche	Candelaria‐Escárcega	Sep–Dec 2020	4860
12	Mexico	Campeche	Escarcega	Mar–Oct 2021	5147
13	Mexico	Campeche	Hecelchakan	Jun–May 2022	1102
14	Mexico	Campeche	Champotón	Sep–Dec 2022	3154
15	Mexico	Campeche	Hecelchakan	Feb–Jun 2023	1479
16	Mexico	Campeche	Calakmul	Sep 2022–Mar 2023	22,231
17	Mexico	Campeche	Calakmul	Apr–Dec 2023	5157
18	Mexico	Campeche	Calakmul	Mar–Jul 2023	2207
19	Mexico	Campeche	Carmen‐Palizada	Feb–May 2024	2733
20	Mexico	Campeche	Escarcega	Oct 2024–Jan 2025	1936
21	Mexico	Yucatán	Tizimin	Feb–Sep 2013	4047
22	Mexico	Yucatán	Tizimin	Apr–Sep 2015	2170
23	Mexico	Yucatán	Oxkutzcab	Nov 2020–Aug 2022	8539
24	Mexico	Yucatán	Tekax	Apr–Oct 2022	2428
25	Mexico	Yucatán	Tizimin	Jan–Nov 2022	3304
26	Mexico	Yucatán	Dzilam de Bravo	Mar 2022–Sep 2024	13,815
27	Mexico	Yucatán	Tizimin	Jan–Dec 2023	3511
28	Mexico	Yucatán	Buczotz	Feb–Dec 2023	3030
29	Mexico	Quintana Roo	Bacalar	May–Nov 2024	4051
30	Guatemala	El Petén	Maya BR, Concesiones Melchor	Apr 2012–Aug 2013	11,073
31	Guatemala	El Petén	Sierra del Lacandón NP	Oct 2014–Oct 2015	14,624
32	Guatemala	El Petén	Maya BR, Buffer zone	Aug–Dec 2017	3780
33	Guatemala	El Progreso	San Agustín Acasaguastlán	May–Jul 2019	267
34	Guatemala	El Petén	Maya BR, Laguna del Tigre NP	Aug–Dec 2019	1849
35	Guatemala	El Petén	Maya BR, Mirador Rio Azul NP	Sep–Dec 2019	2430
36	Guatemala	Zacapa	Usumatlán	May 2019–Jul 2020	2128
37	Guatemala	Zacapa	Usumatlán	Feb‐Oct 2022	1080
38	Guatemala	El Petén	Sierra del Lacandón NP	May–Jul 2023	299
39	Guatemala	El Petén	Maya BR, El Bloque	Jan–May 2024	3076
40	Belize	Orange Walk	Sierra de Agua	Jan–Dec 2017	1525
41	Belize	Orange Walk	Yalbac Area	May–Jul 2019	380
42	Belize	Orange Walk	Hillbank Station	May–Jul 2020	445
43	Belize	Orange Walk	Indian Creek	Jan–Dec 2023	1988
44	Belize	Orange Walk	Bergen's gate	Jan–Dec 2024	1932
				Total	203,682

*Note:* Biosphere reserve is denoted as BR and National Park as NP.

Although these camera trap surveys were designed independently and for different research objectives, together they represent a large cumulative sampling effort distributed across the study region over more than a decade. Camera placement collectively encompassed a wide range of environmental and land‐use contexts, including protected areas, multiple‐use forests, agricultural matrices, peri‐urban landscapes, and human‐modified environments. Cameras were deployed across both remote and accessible sites and over different years and seasons, resulting in broad spatial and temporal coverage that reflects the heterogeneity of landscapes present in the region.

We also consulted publicly accessible online platforms iNaturalist (https://www.inaturalist.org/; iNaturalist.org 2025) and the Global Biodiversity Information Facility (GBIF; http://www.gbif.org; GBIF.org 2025) to compile additional coyote occurrence data from the study region. From these online sources, we obtained coyote occurrence records from the Mexican states of Campeche, Yucatán, and Quintana Roo, as well as in various departments of Guatemala and Belize, which have not been previously published in the scientific literature. We included only occurrence records meeting the following criteria: (i) clear photographic evidence of individual coyotes, either alive or deceased (tracks, scat, or other indirect evidence were excluded), and (ii) data quality classified as “Research Grade” by iNaturalist.

Publicly sourced records complement camera trap data by contributing observations from a broad spectrum of landscape contexts, including rural, agricultural, forested, and protected areas, rather than being restricted to densely populated or urban environments. The integration of structured camera trap surveys with independent public observations increases spatial coverage and reduces reliance on any single data source.

We categorized all records based on their acquisition method and mapped onto a regional distribution map (Figure 1), which also summarizes previously documented coyote records across the study region up to 2021 (see Hidalgo‐Mihart et al. 2022). Detailed information for each record is provided in Table S1.

### 2.3 Habitat Conservation Status of Coyote Recording Sites

We examined the relationship between the coyote occurrence records obtained in this study (excluding previously documented records) and the conservation status of the habitats in which they were recorded.

We utilized the Normalized Difference Vegetation Index (NDVI), a widely used metric for assessing vegetation greenness and stress, which ranges from −1 to 1 (Kriegler et al. 1969). In general, greater NDVI values mean stronger implications for vigorous vegetation greenness: negative values represent water bodies, close to zero for rocks, sands, or concrete surfaces, and positive for vegetation, including crops, shrubs, grasses, and forests (Jones and Vaughan 2010; Huang et al. 2021).

**FIGURE 2:**
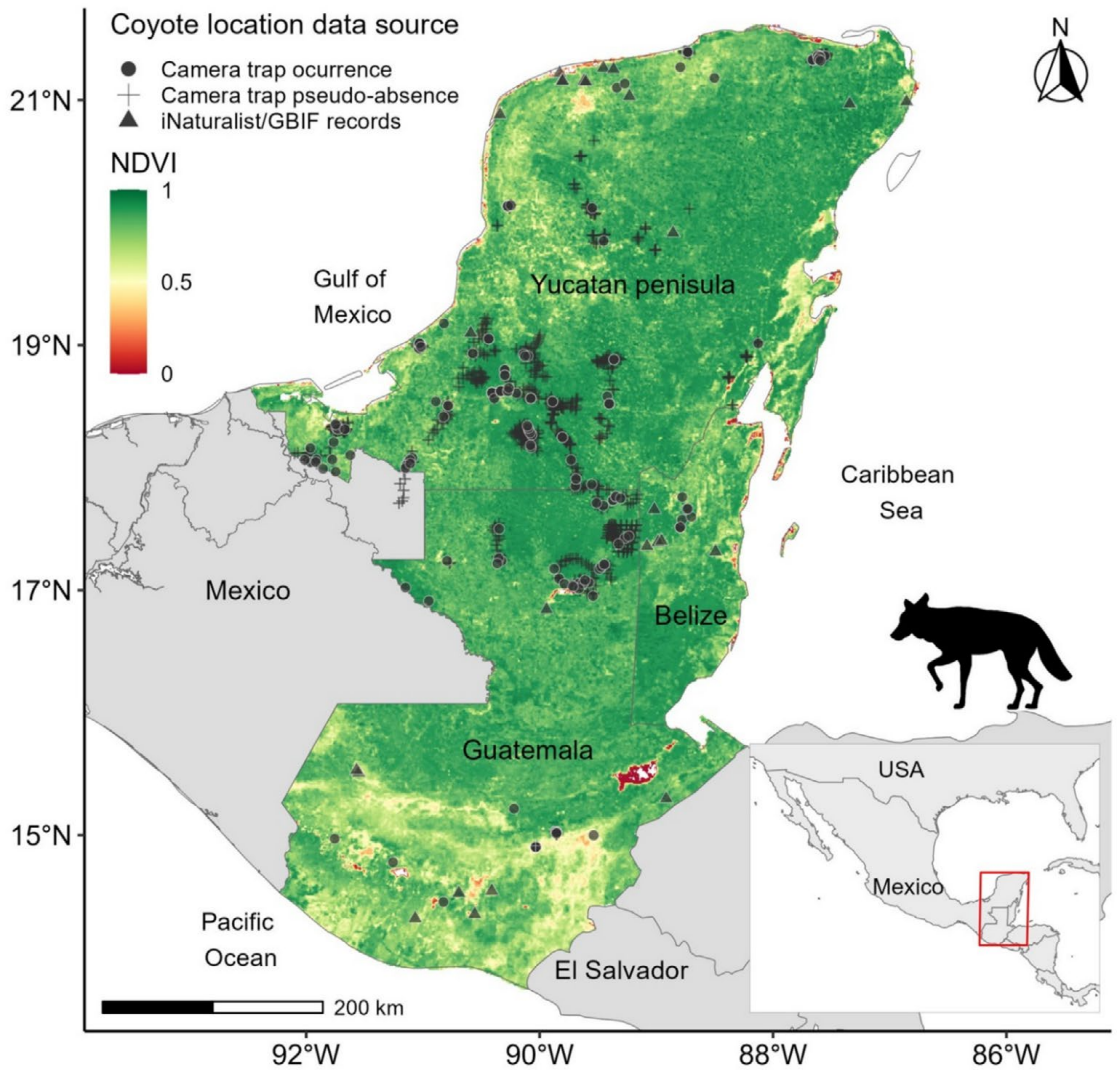
Spatial distribution of the Normalized Difference Vegetation Index (NDVI) across the study region and coyote ( *Canis latrans* ) records in southern Mexico, Guatemala, and Belize. Displayed are camera‐trap detections, camera‐trap pseudo‐absence locations, and records from citizen‐science platforms (iNaturalist and GBIF). Solid lines indicate national boundaries.

The NDVI was selected because it serves as an integrative proxy for habitat heterogeneity, which influences prey availability, cover, and movement opportunities for medium‐sized carnivores such as coyotes. Previous studies have shown that NDVI is strongly associated with carnivore occurrence and space use across heterogeneous landscapes, reflecting both natural and human‐modified habitat conditions (Contreras‐Díaz et al. 2022; Wiegand et al. 2008). In this context, variation in NDVI values captures gradients ranging from open, highly modified areas to more structurally complex vegetated environments that coyotes may exploit opportunistically. We calculated the NDVI data with a resolution of 0.09 km^2^ (Figure 2), from the remote sensing analytical products from the Visible Infrared Imaging Radiometer Suite (eVIIRS) Global NDVI collection (10‐Day Composite, April 1–April 10, 2023), based on the Visible Infrared Imaging Radiometer Suite (VIIRS) provided by the Earth Resources Observation and Science (EROS) Center (Digital Object Identifier: 10.5066/P9QOEFNP), available at https://earthexplorer.usgs.gov/.

**FIGURE 3:**
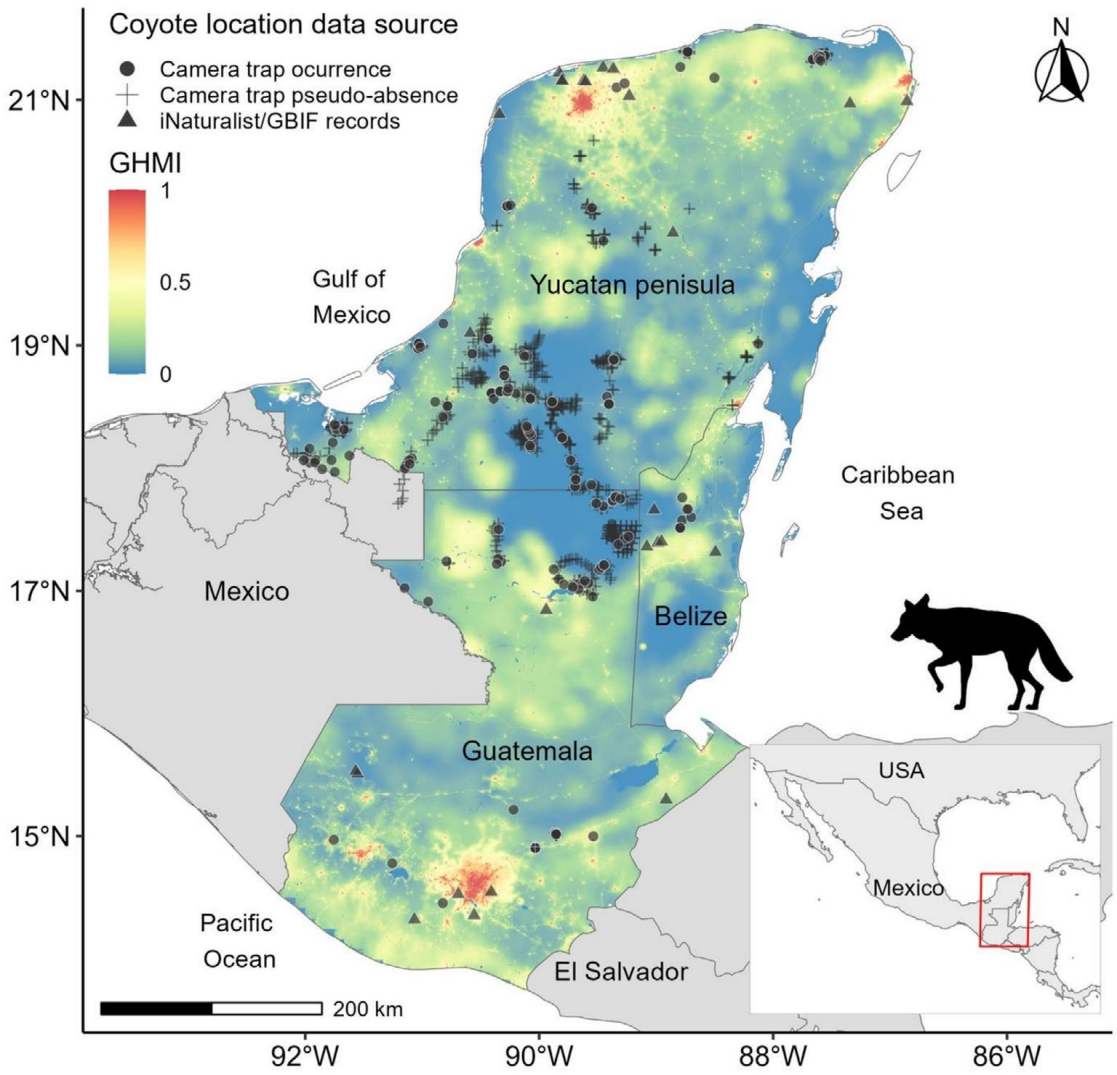
Spatial distribution of the Global Human Modification Index (GHMI) across the study region and coyote ( *Canis latrans* ) records in southern Mexico, Guatemala, and Belize. Shown are camera‐trap detections, camera‐trap pseudo‐absence locations, and records from citizen‐science platforms (iNaturalist and GBIF). Solid lines indicate national boundaries.

Additionally, we used the Global Human Modification Index (GHMI; Kennedy et al. 2019; Theobald et al. 2020), which provides a comprehensive measure of current ecological conditions of the territory and how land cover has been modified (Figure 3). The GHMI evaluates temporal and spatial trends of land use modification of terrestrial lands between 1990~2017, based on spatially explicit modeling of 14 anthropogenic stressors (see Theobald et al. 2020) affecting land cover at a global scale, with a resolution of 0.09 km^2^ (Theobald et al. 2023). The resulting quantitative estimate of the GHMI has values ranging from 0 (unmodified landscapes) to 1 (completely modified landscapes; Kennedy et al. 2019).

We compared the distributions of NDVI and GHMI values for coyote locations with those of the entire study region (Yucatán Peninsula, Guatemala, and Belize) using a two‐sample Kolmogorov–Smirnov test (Conover 1971) at a significance level of *α* = 0.05. Additionally, we generated kernel density distributions for the datasets, interpolated them to align at common evaluation points, and calculated their ratios using the weight function: *w*(*x*) = *p*_nu_(*x*)/*p*_de_(*x*) (Tsuboi et al. 2009), where ‘*w*’ represents the density ratio, ‘*p*_nu_’ denotes the numerator density distribution—corresponding to the NDVI and GHMI at coyote locations—and ‘*p*_de_’ represents the denominator density distribution, referring to the regional NDVI and GHMI values. A ratio > 1 indicates that the density distribution at coyote locations is higher at a given point, a ratio < 1 suggests a higher regional density distribution, and a ratio ≈1 signifies similar densities at that point.

### 2.4 Temporal Trends in Coyote Sampling

To assess temporal trends in sampling effort (camera‐trap days), we implemented a linear regression model (Christensen 2025) using the natural logarithm of sampling effort as the response variable and year as the continuous predictor. The log‐transformation was utilized to satisfy the assumptions of autocorrelation, homoscedasticity, and normality of residuals, which were formally verified through Durbin–Watson (Durbin and Watson 1992), Breusch–Pagan (Breusch and Pagan 1979), and Shapiro–Wilk tests (Royston 1995), respectively. The model was specified as follows:

$$\ln\left({\text{Effort}}_{i}\right)=\beta_{0}+\beta_{1}\left({\text{Year}}_{i}\right)+\varepsilon_{i}$$

where *β*_*0*_ denotes the intercept, *β*_*1*_ the linear effect of year, and *ε*_*i*_ the stochastic error term.

### 2.5 Drivers of Coyote Colonization

To investigate the drivers of coyote occurrence, we fitted a generalized linear model (GLM) with a binomial error distribution and a logit link function (McCullagh and Nelder 1989). The presence‐absence (1/0) of coyotes at monitoring stations was modeled as a function of anthropogenic influence (GHMI), environmental productivity (NDVI) – as a proxy of anthropogenic disturbance–, and spatiotemporal factors. Specifically, the model included the GHMI and the NDVI as continuous environmental predictors, while year and country of detections were incorporated to account for temporal trends and regional variation, respectively.

The model was specified as follows:

$$\begin{array}{c} \text{logit}\left(P_{i}\right)=\ln\left(P_{i}/1-P_{i}\right)=\beta_{0}+\beta_{1}\left({\text{GHMI}}_{i}\right)\\ +\beta_{2}\left({\text{NDVI}}_{i}\right)+\beta_{3}\left({\text{Year}}_{i}\right)+\beta_{4}\left({\text{Country}}_{i}\right)+\varepsilon_{i} \end{array}$$

In this case, *P*_*i*_ represents the probability of coyote occurrence at site *i*, *β*_*0*_ denotes the intercept, and *β*_1‐4_ represent the estimated coefficients for each predictor.

Multicollinearity among predictor variables was assessed using the variance inflation factor (VIF; Fildes 1993; Shrestha 2020), which indicated no significant correlation between variables (VIF≈1). To evaluate the model's discriminative capacity, we calculated the Area Under the Receiver Operating Characteristic (ROC) Curve (AUC; Fawcett 2006). We interpreted AUC values according to Hosmer et al. (2013): values between 0.7 and 0.8 represent moderate discrimination, while those exceeding 0.8 and 0.9 indicate excellent and outstanding discriminative power, respectively.

All statistical analyses were conducted in R software v.4.4.1 (R Core Team 2024). Linear models were implemented using the base *stats* package, while model diagnostics and performance were evaluated using the *lmtest* (Zeileis and Hothorn 2002) and *pROC* (Robin et al. 2011) packages.

## 3 Results

We documented a total of 278 coyote records across the study region, including many from areas where prior evidence of their presence was lacking (Figures 1, 2, 3). Of these, 252 records (90.6%) were obtained from 139 camera‐trap stations (12.4% of the 1125 deployed during 2012–2025). Sampling effort is summarized in Table 1, and detailed location and date information for each record is provided in Table S1. An additional 26 records (9.35%) were retrieved from the online platforms iNaturalist and GBIF.

Among the camera‐trap records, 69.8% (*n* = 176) were collected in Mexico, including 152 from Campeche, 23 from Yucatán, and 1 from Quintana Roo. A further 26.6% (*n* = 67) were recorded in Guatemala, and 3.6% (*n* = 9) in Belize. The earliest documented coyote occurrences in the Yucatán Peninsula and Guatemala date back to May 2013, while the most recent records for both regions were from December 2024. In Belize, the earliest recorded occurrence was in July 2017, and the most recent in July 2024. Regarding records obtained from online platforms, 50% (*n* = 13) were from Mexico, 30.8% (*n* = 8) from Guatemala, and 19.2% (*n* = 5) from Belize. Comprehensive details for each record—including country, state/department, region, date, geographic coordinates, and data source—are provided in the Appendix section (Table S1).

**FIGURE 4:**
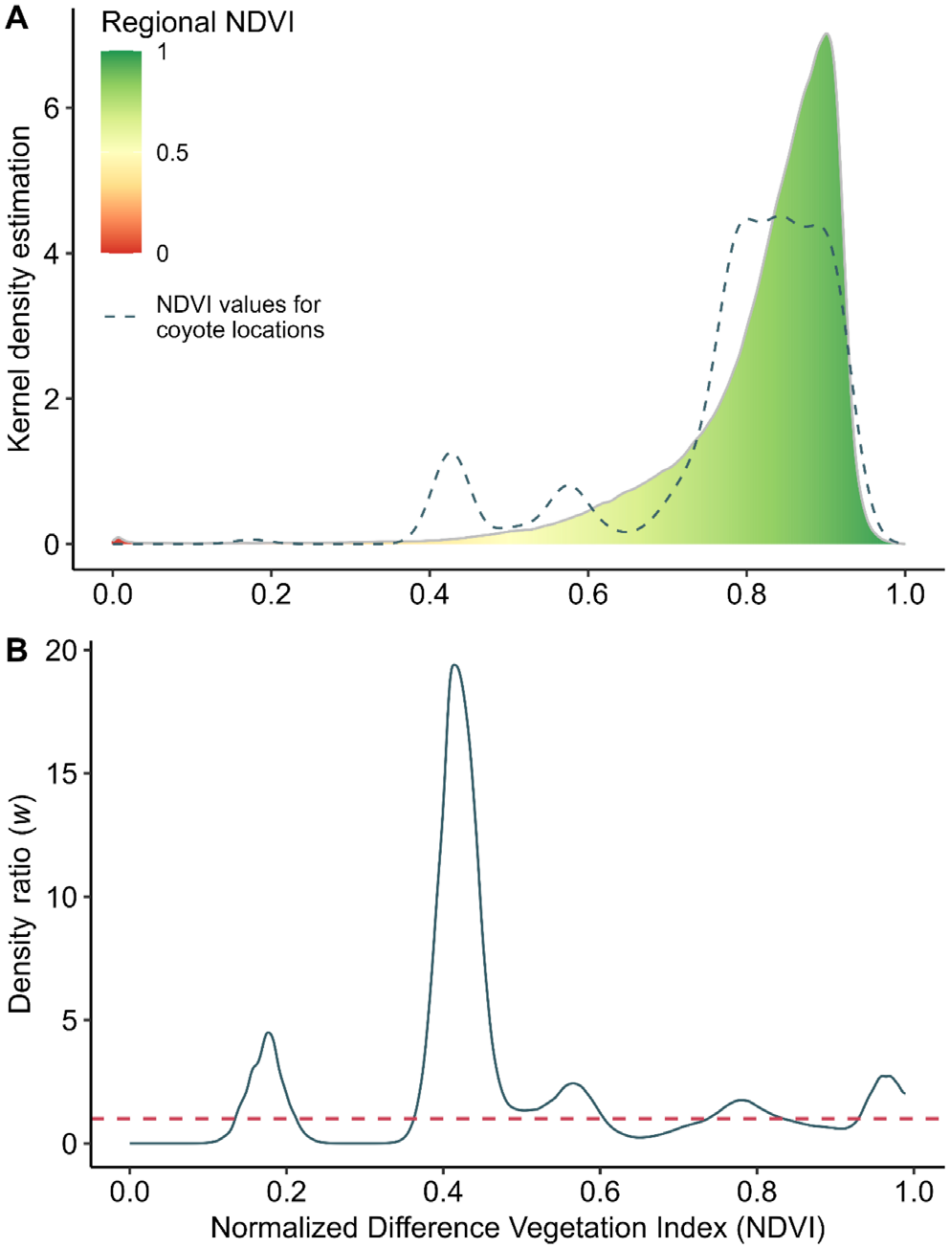
Density distributions for the values of the Normalized Difference Vegetation Index (NDVI). (A) Study region (background continuous colored density; *n* = 3,012,994) and coyote ( *Canis latrans* ) occurrence records in southern Mexico, Guatemala, and Belize (dashed line; *n* = 278). (B) Density ratio: NDVI coyote occurrence distribution/regional NDVI.

**FIGURE 5:**
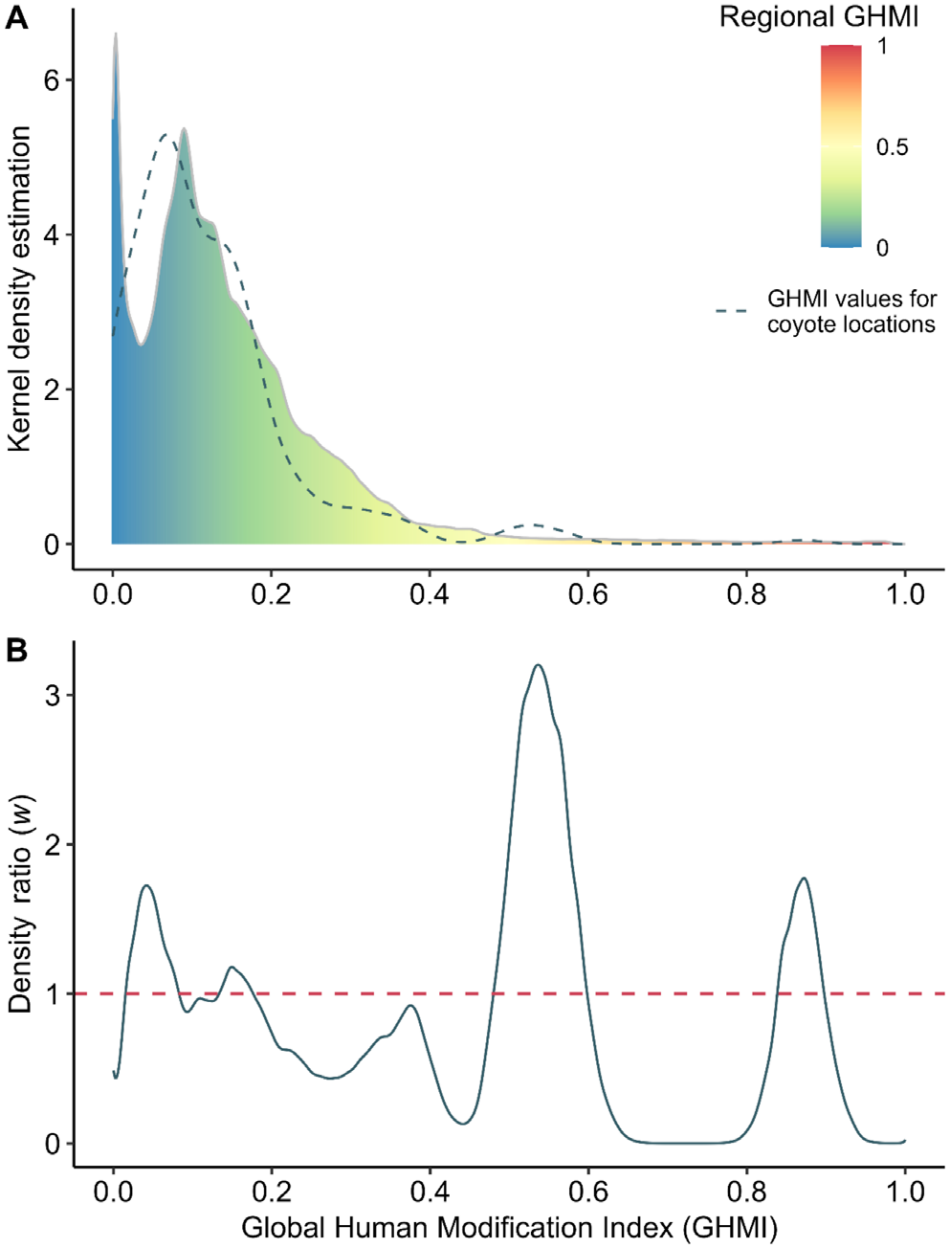
Density distributions for the values of the Global Human Modification Index (GHMI). (A) Study region (background continuous colored density; *n* = 3,140,118) and coyote ( *Canis latrans* ) occurrence records in southern Mexico, Guatemala, and Belize (dashed line; *n* = 278). (B) Density ratio: GHMI coyote occurrence distribution/regional GHMI.

**FIGURE 6:**
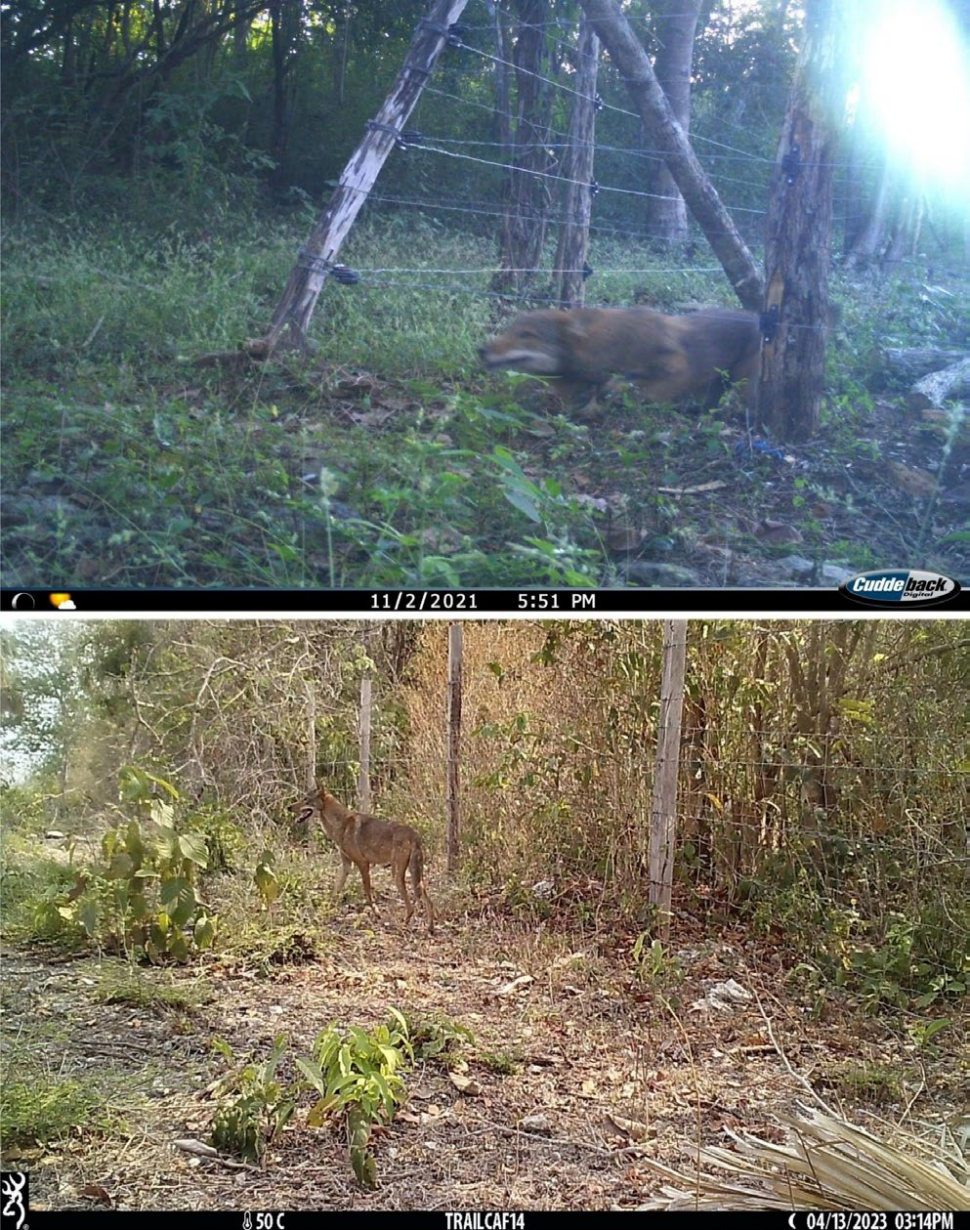
Camera trap records of coyotes ( *Canis latrans* ) in anthropogenically altered landscapes—specifically livestock areas—in Yucatán, Mexico, during 2021 (top) and 2023 (bottom).

The distribution functions of NDVI and GHMI values for the entire region (*n* = 3,012,994 and *n* = 3,140,118, respectively) differed significantly from those associated with coyote locations (*n* = 278; Figures 4 and 5) within the study region (Kolmogorov–Smirnov test: *D* = 0.17, *p* < 0.001; and *D* = 0.16, *p* < 0.001, respectively). Density ratios derived from kernel density estimation showed that coyote occurrences were concentrated in landscapes characterized by low to intermediate vegetation greenness, corresponding to NDVI values typical of open habitats, secondary vegetation, agricultural mosaics, and forest edges, rather than closed‐canopy tropical forest (Figure 4). A secondary peak at intermediate NDVI values (0.4–0.6) indicates frequent use of heterogeneous landscapes where patches of woody vegetation are interspersed with open or managed areas. For human modification, coyote occurrences were most frequent in areas spanning low to high GHMI values, with pronounced peaks at low human influence, moderately modified landscapes, and highly transformed areas (Figure 5). This multimodal pattern suggests that coyotes occupy a broad gradient of land‐use intensity, from relatively low‐impact rural settings to landscapes with substantial anthropogenic infrastructure (Figure 6). Summary statistics of central tendency for NDVI and GHMI values across the entire region and at coyote locations are presented in Table 2.

**TABLE 2:** Central tendency metrics of the Normalized Difference Vegetation Index (NDVI) and the Global Human Modification Index (GHMI) values for the study region and coyote ( *Canis latrans* ) locations in southern Mexico, Guatemala, and Belize.

Parameter	NDVI		GHMI	
	Regional scale	Coyote locations	Regional scale	Coyote locations
Minimum	0	0.17	0	0.01
1st quartile	0.78	0.77	0.06	0.06
Median	0.85	0.82	0.12	0.09
Mean	0.82	0.78	0.15	0.12
3rd quartile	0.89	0.88	0.19	0.15
Maximum	1.00	0.94	0.99	0.87
SD	0.11	0.14	0.13	0.11

Sampling effort remained stable throughout the study period, with no significant temporal trend observed (*p* = 0.77; Figures S1 and S2). The linear model indicated that year was not a significant predictor of the log‐transformed sampling effort (*β*_*1*_ = −0.01, SE = 0.04, *p* = 0.77). These results confirm that the sampling intensity was consistent across years, ensuring that subsequent occurrence analyzes are not confounded by systematic temporal variations in effort.

**FIGURE 7:**
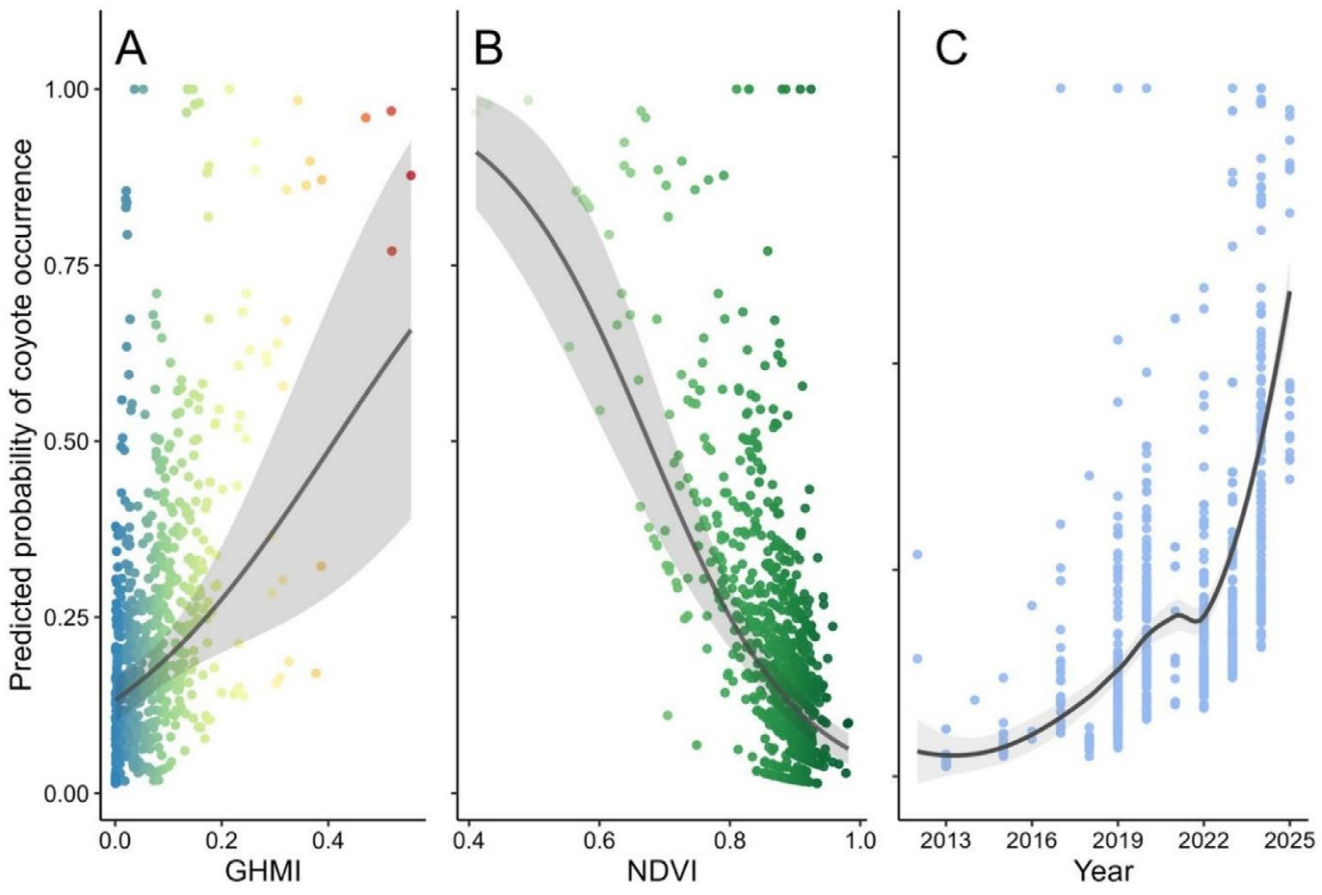
Predicted probability of coyote ( *Canis latrans* ) occurrence based on a generalized linear model (GLM) with a binomial error distribution and a logit link function. Presence–absence (1/0) at monitoring stations was modeled as a function of anthropogenic influence (Global Human Modification Index, GHMI), environmental productivity (Normalized Difference Vegetation Index, NDVI), and spatiotemporal factors. Panels show model‐predicted relationships with (A) GHMI, (B) NDVI, and (C) sampling year. Points represent predicted values for individual records, solid lines indicate fitted model relationships, and shaded areas denote 95% confidence intervals. Country was included in the model to account for regional variation.

The GLM exhibited high discriminative power in distinguishing between coyote presence and absence locations (AUC = 0.83; 95% CI: 0.80–0.86; Figure S2). Coyote occurrence was strongly driven by anthropogenic modification, primary productivity, and temporal factors (Table 3; Figure 7). We found a significant positive association between coyote occurrences and the GHMI (*β* = 4.57, SE = 1.23, *p* < 0.001). Conversely, NDVI—serving as a proxy for habitat openness or anthropogenic disturbance—exerted a strong negative effect (*β* = −8.78, SE = 1.14, *p* < 0.001). Furthermore, a significant positive temporal trend indicated that the probability of occurrence increased annually (*β* = 0.26, SE = 0.03, *p* < 0.001). Regional differences between countries were not statistically significant (*p* > 0.05; Table 3).

**TABLE 3:** Determinants of coyote ( *Canis latrans* ) occurrence. Odds ratios (OR), 95% confidence intervals (95% CI), and *p* values are derived from a generalized linear model including anthropogenic (GHMI), vegetation (NDVI), and spatiotemporal covariates (year and country).

Variable	log(OR)	95% CI	*p*
GHMI	4.57	2.18, 7.01	< 0.001
NDVI	−8.78	−11.08, −6.63	< 0.001
Year	0.26	0.20, 0.34	< 0.001
Country			
Belize	−511.89	—	0.27
Guatemala	−16.81	—	0.97
Mexico	−17.05	—	0.97

## 4 Discussion

Our findings provide substantial new evidence of coyote presence in southeastern Mexico, Guatemala, and Belize, significantly expanding the known distribution of the species in these regions. We reveal that coyotes have successfully established in human‐modified tropical landscapes over just the past decade. These insights are supported by an extensive sampling effort exceeding 200,000 camera‐trap days conducted over a 12‐year period (2012–2024), lending strong empirical support to the observed patterns.

While previous studies have documented similar expansions in temperate regions—such as the northeastern United States and Canada (Gompper 2002; Chubbs and Phillips 2005; Fener et al. 2005; Kays et al. 2009) and have modeled potential expansion across North and Central America more broadly (Ellington and Murray 2015; Hody and Kays 2018; Monroy‐Vilchis et al. 2020), our results provide direct empirical evidence of coyote range expansion in tropical landscapes. Notably, we document coyote occurrence in regions such as the Yucatán Peninsula and parts of Guatemala that were previously predicted to have low suitability (Monroy‐Vilchis et al. 2020), highlighting the limitations of distribution models calibrated on historical records and underscoring the dynamic nature of species distributions in rapidly human‐modified tropical environments.

Our results suggest that habitat modification and land‐use change are among the most plausible drivers of coyote expansion in the Yucatán Peninsula and adjacent regions. As documented in northern North America, the conversion of continuous forest into agricultural and urban mosaics can favor coyotes by increasing habitat heterogeneity and prey availability (Hidalgo‐Mihart et al. 2004; Hody and Kays 2018). Consistent with this pattern, many of our records occurred in areas with strong human influence—particularly agricultural and urbanized landscapes in northern Yucatán and Quintana Roo—supporting the hypothesis that anthropogenic disturbance facilitates coyote colonization of new areas (Vaughan 1983; Cove et al. 2012).

Previous work suggested that coyotes were largely absent from regions dominated by extensive tracts of tropical moist forest in southern Mexico and Central America, including much of the Yucatán Peninsula (Hidalgo‐Mihart et al. 2004). However, land‐use dynamics in the region have changed substantially over recent decades. In Quintana Roo alone, more than 330,000 ha of mature forest were converted to induced pastures and secondary vegetation between 2000 and 2013, particularly in the southern portion of the state (Ellis et al. 2017), but also in the northeastern region where forest loss has been driven primarily by rapid and largely unplanned urban expansion (Ellis et al. 2017). The resulting transformation of closed‐canopy forest into more open and fragmented habitats likely reduces ecological barriers to coyote establishment and may continue to promote their expansion as land‐use change progresses.

Coyotes are not the only mammal species whose recent range expansion in the eastern Yucatán Peninsula appears to be associated with deforestation. Hidalgo‐Mihart et al. (2017) documented the expansion of the eastern cottontail rabbit ( *Sylvilagus floridanus* ) in Quintana Roo, likely linked to land‐use change. The concurrent expansion of multiple species adapted to open or human‐modified habitats suggests that broader faunal turnover may be underway. This underscores the need for increased vertebrate monitoring in deforested and rapidly transforming landscapes to detect early colonization events and inform timely management actions aimed at mitigating the spread of potentially invasive species.

The occurrence of coyotes across both natural and anthropogenically altered landscapes further suggests that population expansion is ongoing throughout the region. As populations grow, dispersal of juvenile individuals seeking new territories is likely to increase the frequency of records across a wide range of habitat conditions, including both well‐preserved forest remnants and human‐modified environments. Evidence from other systems indicates that early life exposure to human‐modified habitats can influence dispersal behavior, with individuals originating from more developed natal home ranges being more likely to disperse and to do so over greater distances than those raised in more natural habitats (Zepeda et al. 2021). This mechanism provides a plausible explanation for the continued spread of coyotes into heterogeneous tropical landscapes, where dispersing individuals from anthropogenically influenced areas may facilitate colonization across diverse habitat contexts.

The expansion of coyotes into the Maya Forest region exemplifies how adaptable carnivores can exploit human‐altered landscapes. This finding aligns with broader patterns reported for other mesopredators globally, where anthropogenic transformation not only reduces the abundance of larger competitors but also increases access to predictable resources such as refuse, domestic animals, and synanthropic prey species (Bateman and Fleming 2012; Gehrt et al. 2009). Unlike their northern expansion, where the extirpation of apex predators such as wolves ( *Canis lupus* ) has been a significant factor (Gompper 2002; Berger and Gese 2007; Newsome and Ripple 2015), coyotes in the Yucatán Peninsula coexist with robust populations of jaguars and mountain lions (Chávez et al. 2007; Ceballos et al. 2021). This context suggests that coyote expansion in tropical Mesoamerica is not primarily explained by mesopredator release, but rather by the ability of coyotes to persist within heterogeneous and human‐modified landscapes.

Behavioral plasticity and dietary generalism have been proposed as key traits facilitating coyote expansion across diverse environments (Andelt et al. 1987; Kitchen et al. 2000; Rodríguez‐Luna et al. 2021, 2024; Jensen et al. 2022). While our study does not directly test these mechanisms, the observed associations between coyote occurrence, human modification, and vegetation structure are consistent with these traits enabling landscape‐level flexibility. This behavioral plasticity (Andelt et al. 1987; Kitchen et al. 2000; Rodríguez‐Luna et al. 2024) and dietary generalism (Rodríguez‐Luna et al. 2021; Jensen et al. 2022) may allow coyotes to navigate novel ecological contexts where apex predators persist (Arjo and Pletscher 1999; Berger and Gese 2007).

Another potential factor contributing to coyote expansion is behavioral plasticity, which parallels mechanisms proposed for invasive species, where heterospecific associations may buffer against low conspecific density during early stages of range expansion (Camacho‐Cervantes et al. 2023). In human‐modified landscapes, increasing spatial overlap between coyotes and domestic dogs may facilitate such heterospecific interactions, potentially providing social or ecological benefits during colonization. In this context, hybridization—documented in northeastern populations where coyotes have interbred with wolves and domestic dogs, resulting in genotypes that may enhance adaptability to novel habitats (Kays et al. 2009; Thornton and Murray 2014; Ellington and Murray 2015)—may represent one outcome of sustained coyote–dog interfaces. Beyond genetic consequences, these interfaces may also have ecological and epidemiological implications, as coyotes and dogs can share parasites and pathogens in human‐modified environments, potentially influencing survival, movement, and persistence (Monge‐Nájera and Brenes 1987; Marcek et al. 2023). While the roles of heterospecific socialization, hybridization, and shared disease dynamics remain largely untested in southeastern Mesoamerica, these processes together represent promising avenues for future research aimed at understanding the mechanisms underlying coyote persistence and expansion in tropical regions.

The ecological impacts of coyote expansion into southeastern Mexico, Guatemala, and Belize are similar to those recognized for invasive species (Carneiro et al. 2025). Coyotes could be influencing community structure through predation and competition, potentially reshaping predator guilds and ecosystem function where they now overlap with species like jaguars, mountain lions, and ocelots ( *Leopardus pardalis* ), as in temperate ecosystems where coyote colonization alters interactions with larger predators like mountain lions and wolves (Wang et al. 2015; Crosby et al. 2024). At present, however, direct evidence for such community‐level effects in tropical Mesoamerica is limited, and further work is needed to assess impacts on native carnivores, prey communities, and ecosystem processes. Comparisons with other expanding canids, such as the golden jackal ( *Canis aureus* ) in Europe (Krofel et al. 2017), provide useful analogs but should be interpreted cautiously given ecological and biogeographic differences.

In contrast to the core areas of their range, where human‐coyote conflicts are common (Kays et al. 2015; Baker and Timm 2017; Davenport et al. 2022; Raymond and St. Clair 2023; Torres‐Romero et al. 2023; Bradfield et al. 2025), coyotes in newly colonized areas of Mesoamerica remain at low densities and show limited habituation to people, reducing the likelihood of conflict for now (White and Gehrt 2009). Nevertheless, early signs of misattributed livestock predation, particularly where coyote kills are mistaken for jaguar attacks, highlight how their presence could complicate large carnivore conservation (Contreras‐Moreno, pers. comm.). Recent reports from the Yucatán Peninsula confirm incidents of coyote predation on livestock (Torres‐Romero et al. 2023), suggesting that conflict potential may increase as populations grow.

This situation presents a critical window for proactive and culturally grounded management. Experiences from other regions highlight that successful wildlife conflict prevention depends not only on early intervention but also on education and integration of local cultural values, traditional ecological knowledge, and inclusive governance. For example, in Denver and Edmonton, community‐level education, hazing programs, and monitoring systems have proven effective when deployed before conflicts intensify (Lukasik and Alexander 2011; Gehrt et al. 2009; Baker et al. 2020). Similarly, indigenous wildlife management in Morelos, Mexico (Bello‐Román et al. 2023) and community‐based conservation in Belize (Horwich and Lyon 2007) have demonstrated how traditional knowledge systems can contribute to conservation and coexistence. As coyotes continue expanding in Mesoamerica, engaging rural, indigenous, and urban communities through culturally appropriate strategies will be key to fostering coexistence while safeguarding broader biodiversity goals.

This case study also invites reflection on the conceptual distinction between native species range expansions and biological invasions. Both processes involve the spread of organisms into previously unoccupied areas, but their framing differs in biogeographic and management contexts (Blackburn et al. 2011). Coyotes are native to North America but have expanded into regions where they were historically absent, driven in part by anthropogenic change. As Bocedi et al. (2014) emphasize, species' range limits are dynamic and shaped by interactions between dispersal behavior, landscape structure, and ecological filters. This blurring of boundaries challenges strict dichotomies between “native” and “invasive” and reinforces the need to view range dynamics as part of broader Anthropocene biogeographical change (Storch et al. 2021).

Despite the extensive and geographically distributed sampling effort, several limitations should be acknowledged. Although the cumulative effort is sufficient to capture a realistic probability of detecting coyotes across contrasting land‐use contexts, some degree of spatial bias inherent to opportunistic, project‐driven, or accessibility‐constrained sampling cannot be entirely excluded. Camera‐trap deployments were designed independently across projects and varied in objectives, spatial coverage, and intensity, which may influence local detection probabilities. Nevertheless, the diversity of survey designs, locations, and sampling intensities, combined with the inclusion of both detections and non‐detections, reduces the likelihood that occurrence records are disproportionately derived from either strictly protected areas or heavily human‐dominated landscapes. Additionally, our analyzes relied on broadscale environmental proxies (GHMI and NDVI), which capture major gradients of human modification and vegetation structure but cannot resolve finer scale habitat features or behavioral mechanisms influencing coyote presence. Future studies integrating standardized sampling designs, movement data, and finer resolution ecological and social variables would help refine understanding of the processes underlying coyote expansion in tropical Mesoamerica.

## Author Contributions

**César R. Rodríguez‐Luna:** conceptualization (lead), data curation (lead), formal analysis (lead), investigation (equal), methodology (equal), resources (equal), validation (equal), visualization (equal), writing – original draft (equal), writing – review and editing (equal). **Fernando M. Contreras‐Moreno:** data curation (equal), investigation (equal), methodology (equal), resources (equal), writing – review and editing (supporting). **Morelia Camacho‐Cervantes:** investigation (supporting), writing – original draft (equal), writing – review and editing (equal). **Daniel Jesús‐Espinosa:** data curation (supporting), investigation (supporting), methodology (supporting), resources (supporting). **Luis A. Trujillo‐Sosa:** data curation (supporting), investigation (supporting), resources (supporting). **Alma C. Escobar‐Cifuentes:** data curation (supporting), investigation (supporting), resources (supporting). **Alejandro Marmol:** data curation (supporting), investigation (supporting), resources (supporting). **Rony García‐Anleu:** data curation (equal), investigation (equal), resources (equal). **Martha P. Ibarra‐López:** data curation (supporting), investigation (equal), methodology (equal), resources (supporting). **Román Espinal‐Palomino:** data curation (supporting), methodology (supporting), visualization (equal), writing – review and editing (equal). **Anuar D. Hernández‐SaintMartín:** data curation (equal), investigation (equal), resources (equal). **Patricio Canul‐Chuc:** data curation (supporting), resources (supporting). **Víctor Castelazo‐Calva:** data curation (supporting), methodology (supporting), resources (supporting), validation (supporting). **Marcos Corado:** data curation (supporting), investigation (supporting), resources (supporting). **Alberto González‐Gallina:** data curation (equal), investigation (equal), resources (equal). **Pedro E. Nahuat‐Cervera:** data curation (equal), investigation (equal). **Mircea G. Hidalgo‐Mihart:** data curation (equal), investigation (equal), methodology (equal), resources (equal), writing – review and editing (equal). **Carlos N. Ibarra‐Cerdeña:** conceptualization (lead), funding acquisition (lead), investigation (lead), methodology (lead), project administration (lead), resources (lead), supervision (lead), validation (lead), writing – original draft (lead), writing – review and editing (lead).

## Conflicts of Interest

The authors declare no conflicts of interest.

## Supporting information


**Table S1:** ece373184‐sup‐0001‐TableS1.docx.

**Figures S1–S2:** ece373184‐sup‐0002‐FigureS1‐S2.docx.

## Data Availability

The data (Rodríguez‐Luna et al. 2025) supporting the findings of this study are available from the Figshare Digital Repository: https://doi.org/10.6084/m9.figshare.29068625.
