# Supplementary material for: Coyote Range Expansion in the Human‐Modified Tropics of Mesoamerica
Source: Ecol Evol. 2026 Mar 2;16(3):e73184. doi: 10.1002/ece3.73184 (PMC12953002; doi:10.1002/ece3.73184)
Supplement: Supplementary file 1 — Table S1: ece373184‐sup‐0001‐TableS1.docx. [file ECE3-16-e73184-s001.docx]

**Appendix A**

TABLE A1. Detailed information of the occurrence records of coyotes (*Canis latrans*) across southern Mexico, Guatemala, and Belize: country, state/department, region, date, geographic coordinates, and data source.

| **ID** | **Country** | **State/Department** | **Region** | **Species** | **Date** | **DDN** | **DDW** | **Source** |
| --- | --- | --- | --- | --- | --- | --- | --- | --- |
| 1 | Mexico | Campeche | Escarcega | *Canis latrans* | 2024/12/19 | -90.29792 | 18.7538 | Cámera trap survey |
| 2 | Mexico | Campeche | Escarcega | *Canis latrans* | 2024/12/19 | -90.29792 | 18.7538 | Cámera trap survey |
| 3 | Mexico | Campeche | Escarcega | *Canis latrans* | 2024/10/29 | -90.29887 | 18.79773 | Cámera trap survey |
| 4 | Mexico | Campeche | Escarcega | *Canis latrans* | 2024/10/27 | -90.29887 | 18.79773 | Cámera trap survey |
| 5 | Mexico | Campeche | Nicte Ha | *Canis latrans* | 2024/06/02 | -91.91834 | 18.0462 | Cámera trap survey |
| 6 | Mexico | Campeche | Nicte Ha | *Canis latrans* | 2024/05/02 | -92.01243 | 18.06311 | Cámera trap survey |
| 7 | Mexico | Campeche | Nicte Ha | *Canis latrans* | 2024/02/07 | -91.96281 | 18.04433 | Cámera trap survey |
| 8 | Mexico | Campeche | Nicte Ha | *Canis latrans* | 2024/02/06 | -91.91171 | 18.06539 | Cámera trap survey |
| 9 | Mexico | Campeche | Calakmul | *Canis latrans* | 2023/06/23 | -89.68782 | 17.9068 | Cámera trap survey |
| 10 | Mexico | Campeche | Justicia Social | *Canis latrans* | 2023/06/10 | -90.40819 | 18.61104 | Cámera trap survey |
| 11 | Mexico | Campeche | Lopez Mateos | *Canis latrans* | 2023/06/02 | -90.33295 | 18.62436 | Cámera trap survey |
| 12 | Mexico | Campeche | Lopez Mateos | *Canis latrans* | 2023/06/02 | -90.33295 | 18.62436 | Cámera trap survey |
| 13 | Mexico | Campeche | Hecelchakan | *Canis latrans* | 2023/05/22 | -90.27302 | 20.13524 | Cámera trap survey |
| 14 | Mexico | Campeche | Hecelchakan | *Canis latrans* | 2023/05/21 | -90.27302 | 20.13524 | Cámera trap survey |
| 15 | Mexico | Campeche | Calakmul | *Canis latrans* | 2023/05/18 | -89.36299 | 18.88289 | Cámera trap survey |
| 16 | Mexico | Campeche | Lopez Mateos | *Canis latrans* | 2023/05/15 | -90.33295 | 18.62436 | Cámera trap survey |
| 17 | Mexico | Campeche | Silvituc | *Canis latrans* | 2023/05/15 | -90.272 | 18.62458 | Cámera trap survey |
| 18 | Mexico | Campeche | Silvituc | *Canis latrans* | 2023/05/15 | -90.272 | 18.62458 | Cámera trap survey |
| 19 | Mexico | Campeche | Justicia Social | *Canis latrans* | 2023/05/12 | -90.40819 | 18.61104 | Cámera trap survey |
| 20 | Mexico | Campeche | Lopez Mateos | *Canis latrans* | 2023/05/10 | -90.33295 | 18.62436 | Cámera trap survey |
| 21 | Mexico | Campeche | Calakmul | *Canis latrans* | 2023/05/07 | -89.68782 | 17.9068 | Cámera trap survey |
| 22 | Mexico | Campeche | Calakmul | *Canis latrans* | 2023/05/07 | -89.69296 | 17.85053 | Cámera trap survey |
| 23 | Mexico | Campeche | Calakmul | *Canis latrans* | 2023/04/28 | -89.73013 | 18.06269 | Cámera trap survey |
| 24 | Mexico | Campeche | Justicia Social | *Canis latrans* | 2023/04/25 | -90.40819 | 18.61104 | Cámera trap survey |
| 25 | Mexico | Campeche | Calakmul | *Canis latrans* | 2023/04/13 | -89.36299 | 18.88289 | Cámera trap survey |
| 26 | Mexico | Campeche | Justicia Social | *Canis latrans* | 2023/04/13 | -90.38885 | 18.5636 | Cámera trap survey |
| 27 | Mexico | Campeche | Lopez Mateos | *Canis latrans* | 2023/04/11 | -90.33295 | 18.62436 | Cámera trap survey |
| 28 | Mexico | Campeche | Justicia Social | *Canis latrans* | 2023/04/10 | -90.40819 | 18.61104 | Cámera trap survey |
| 29 | Mexico | Campeche | Lopez Mateos | *Canis latrans* | 2023/03/24 | -90.33295 | 18.62436 | Cámera trap survey |
| 30 | Mexico | Campeche | Justicia Social | *Canis latrans* | 2023/03/07 | -90.40819 | 18.61104 | Cámera trap survey |
| 31 | Mexico | Campeche | Justicia Social | *Canis latrans* | 2023/03/07 | -90.40819 | 18.61104 | Cámera trap survey |
| 32 | Mexico | Campeche | Justicia Social | *Canis latrans* | 2023/03/03 | -90.40819 | 18.61104 | Cámera trap survey |
| 33 | Mexico | Campeche | Justicia Social | *Canis latrans* | 2023/03/02 | -90.40819 | 18.61104 | Cámera trap survey |
| 34 | Mexico | Campeche | Justicia Social | *Canis latrans* | 2023/02/28 | -90.40819 | 18.61104 | Cámera trap survey |
| 35 | Mexico | Campeche | Lopez Mateos | *Canis latrans* | 2023/02/28 | -90.33295 | 18.62436 | Cámera trap survey |
| 36 | Mexico | Campeche | Justicia Social | *Canis latrans* | 2023/02/27 | -90.40819 | 18.61104 | Cámera trap survey |
| 37 | Mexico | Campeche | Justicia Social | *Canis latrans* | 2023/02/25 | -90.40819 | 18.61104 | Cámera trap survey |
| 38 | Mexico | Campeche | Lopez Mateos | *Canis latrans* | 2023/02/23 | -90.33295 | 18.62436 | Cámera trap survey |
| 39 | Mexico | Campeche | Justicia Social | *Canis latrans* | 2023/02/20 | -90.40819 | 18.61104 | Cámera trap survey |
| 40 | Mexico | Campeche | Lopez Mateos | *Canis latrans* | 2023/02/09 | -90.33295 | 18.62436 | Cámera trap survey |
| 41 | Mexico | Campeche | Calakmul | *Canis latrans* | 2023/01/02 | -89.40422 | 18.52093 | Cámera trap survey |
| 42 | Mexico | Campeche | Calakmul | *Canis latrans* | 2023/01/01 | -89.40103 | 18.52076 | Cámera trap survey |
| 43 | Mexico | Campeche | Calakmul | *Canis latrans* | 2022/12/31 | -89.40258 | 18.52088 | Cámera trap survey |
| 44 | Mexico | Campeche | Calakmul | *Canis latrans* | 2022/12/24 | -89.42046 | 18.58175 | Cámera trap survey |
| 45 | Mexico | Campeche | Balam Ku | *Canis latrans* | 2022/10/06 | -90.43631 | 19.05186 | Cámera trap survey |
| 46 | Mexico | Campeche | Balam Ku | *Canis latrans* | 2022/10/06 | -90.43631 | 19.05186 | Cámera trap survey |
| 47 | Mexico | Campeche | Hecelchakan | *Canis latrans* | 2022/08/22 | -90.27302 | 20.13524 | Cámera trap survey |
| 48 | Mexico | Campeche | Hecelchakan | *Canis latrans* | 2022/08/09 | -90.26316 | 20.13941 | Cámera trap survey |
| 49 | Mexico | Campeche | Calakmul | *Canis latrans* | 2022/07/30 | -89.55313 | 17.86355 | Cámera trap survey |
| 50 | Mexico | Campeche | Hecelchakan | *Canis latrans* | 2022/07/24 | -90.27302 | 20.13524 | Cámera trap survey |
| 51 | Mexico | Campeche | Hecelchakan | *Canis latrans* | 2022/07/21 | -90.27302 | 20.13524 | Cámera trap survey |
| 52 | Mexico | Campeche | Hecelchakan | *Canis latrans* | 2022/07/04 | -90.26316 | 20.13941 | Cámera trap survey |
| 53 | Mexico | Campeche | Escarcega | *Canis latrans* | 2022/07/04 | -90.26378 | 18.64973 | Cámera trap survey |
| 54 | Mexico | Campeche | Hecelchakan | *Canis latrans* | 2022/07/03 | -90.26316 | 20.13941 | Cámera trap survey |
| 55 | Mexico | Campeche | Hecelchakan | *Canis latrans* | 2022/06/25 | -90.27302 | 20.13524 | Cámera trap survey |
| 56 | Mexico | Campeche | Hecelchakan | *Canis latrans* | 2022/06/24 | -90.26316 | 20.13941 | Cámera trap survey |
| 57 | Mexico | Campeche | Hecelchakan | *Canis latrans* | 2022/06/24 | -90.27302 | 20.13524 | Cámera trap survey |
| 58 | Mexico | Campeche | Hecelchakan | *Canis latrans* | 2022/06/17 | -90.27302 | 20.13524 | Cámera trap survey |
| 59 | Mexico | Campeche | Hecelchakan | *Canis latrans* | 2022/06/17 | -90.27302 | 20.13524 | Cámera trap survey |
| 60 | Mexico | Campeche | Hecelchakan | *Canis latrans* | 2022/06/09 | -90.27302 | 20.13524 | Cámera trap survey |
| 61 | Mexico | Campeche | Hecelchakan | *Canis latrans* | 2022/06/04 | -90.27302 | 20.13524 | Cámera trap survey |
| 62 | Mexico | Campeche | Hecelchakan | *Canis latrans* | 2022/06/03 | -90.27302 | 20.13524 | Cámera trap survey |
| 63 | Mexico | Campeche | Hecelchakan | *Canis latrans* | 2022/05/28 | -90.27302 | 20.13524 | Cámera trap survey |
| 64 | Mexico | Campeche | Hecelchakan | *Canis latrans* | 2022/05/26 | -90.27302 | 20.13524 | Cámera trap survey |
| 65 | Mexico | Campeche | Hecelchakan | *Canis latrans* | 2022/05/23 | -90.27302 | 20.13524 | Cámera trap survey |
| 66 | Mexico | Campeche | Hecelchakan | *Canis latrans* | 2022/05/23 | -90.27302 | 20.13524 | Cámera trap survey |
| 67 | Mexico | Campeche | Hecelchakan | *Canis latrans* | 2022/04/29 | -90.27302 | 20.13524 | Cámera trap survey |
| 68 | Mexico | Campeche | Hecelchakan | *Canis latrans* | 2022/04/29 | -90.27302 | 20.13524 | Cámera trap survey |
| 69 | Mexico | Campeche | Hecelchakan | *Canis latrans* | 2022/04/25 | -90.26316 | 20.13941 | Cámera trap survey |
| 70 | Mexico | Campeche | Hecelchakan | *Canis latrans* | 2022/04/25 | -90.27302 | 20.13524 | Cámera trap survey |
| 71 | Mexico | Campeche | Hecelchakan | *Canis latrans* | 2022/04/24 | -90.27302 | 20.13524 | Cámera trap survey |
| 72 | Mexico | Campeche | Hecelchakan | *Canis latrans* | 2022/04/23 | -90.26316 | 20.13941 | Cámera trap survey |
| 73 | Mexico | Campeche | Hecelchakan | *Canis latrans* | 2022/04/23 | -90.27302 | 20.13524 | Cámera trap survey |
| 74 | Mexico | Campeche | Hecelchakan | *Canis latrans* | 2022/04/11 | -90.26316 | 20.13941 | Cámera trap survey |
| 75 | Mexico | Campeche | Hecelchakan | *Canis latrans* | 2022/04/04 | -90.26316 | 20.13941 | Cámera trap survey |
| 76 | Mexico | Campeche | Hecelchakan | *Canis latrans* | 2022/04/04 | -90.27302 | 20.13524 | Cámera trap survey |
| 77 | Mexico | Campeche | Hecelchakan | *Canis latrans* | 2022/04/02 | -90.26316 | 20.13941 | Cámera trap survey |
| 78 | Mexico | Campeche | Hecelchakan | *Canis latrans* | 2022/03/29 | -90.26316 | 20.13941 | Cámera trap survey |
| 79 | Mexico | Campeche | Hecelchakan | *Canis latrans* | 2022/03/29 | -90.27302 | 20.13524 | Cámera trap survey |
| 80 | Mexico | Campeche | Hecelchakan | *Canis latrans* | 2022/03/29 | -90.27302 | 20.13524 | Cámera trap survey |
| 81 | Mexico | Campeche | Hecelchakan | *Canis latrans* | 2022/03/24 | -90.27302 | 20.13524 | Cámera trap survey |
| 82 | Mexico | Campeche | Hecelchakan | *Canis latrans* | 2022/03/23 | -90.27302 | 20.13524 | Cámera trap survey |
| 83 | Mexico | Campeche | Hecelchakan | *Canis latrans* | 2021/12/27 | -90.27302 | 20.13524 | Cámera trap survey |
| 84 | Mexico | Campeche | Hecelchakan | *Canis latrans* | 2021/12/23 | -90.27302 | 20.13524 | Cámera trap survey |
| 85 | Mexico | Campeche | Hecelchakan | *Canis latrans* | 2021/11/26 | -90.27302 | 20.13524 | Cámera trap survey |
| 86 | Mexico | Campeche | Hecelchakan | *Canis latrans* | 2021/11/22 | -90.26316 | 20.13941 | Cámera trap survey |
| 87 | Mexico | Campeche | Hecelchakan | *Canis latrans* | 2021/11/14 | -90.26316 | 20.13941 | Cámera trap survey |
| 88 | Mexico | Campeche | Hecelchakan | *Canis latrans* | 2021/11/07 | -90.26316 | 20.13941 | Cámera trap survey |
| 89 | Mexico | Campeche | Hecelchakan | *Canis latrans* | 2021/11/05 | -90.26316 | 20.13941 | Cámera trap survey |
| 90 | Mexico | Campeche | Pomuch | *Canis latrans* | 2021/11/02 | -90.24662 | 20.14307 | Cámera trap survey |
| 91 | Mexico | Campeche | Hecelchakan | *Canis latrans* | 2021/09/18 | -90.27302 | 20.13524 | Cámera trap survey |
| 92 | Mexico | Campeche | División del Norte | *Canis latrans* | 2020/11/19 | -90.78432 | 18.50525 | Cámera trap survey |
| 93 | Mexico | Campeche | División del Norte | *Canis latrans* | 2020/11/09 | -90.78432 | 18.50525 | Cámera trap survey |
| 94 | Mexico | Campeche | Don Samuel | *Canis latrans* | 2020/11/08 | -90.82393 | 18.41544 | Cámera trap survey |
| 95 | Mexico | Campeche | El Naranjo | *Canis latrans* | 2020/11/04 | -91.14903 | 17.99659 | Cámera trap survey |
| 96 | Mexico | Campeche | El Naranjo | *Canis latrans* | 2020/10/31 | -91.13131 | 18.03479 | Cámera trap survey |
| 97 | Mexico | Campeche | El Naranjo | *Canis latrans* | 2020/10/23 | -91.14281 | 18.01723 | Cámera trap survey |
| 98 | Mexico | Campeche | El Naranjo | *Canis latrans* | 2020/10/22 | -91.12263 | 18.05091 | Cámera trap survey |
| 99 | Mexico | Campeche | Paraiso Nuevo | *Canis latrans* | 2020/10/04 | -91.0896 | 18.07977 | Cámera trap survey |
| 100 | Mexico | Campeche | Pixoyal | *Canis latrans* | 2020/10/01 | -90.57235 | 18.93228 | Cámera trap survey |
| 101 | Mexico | Campeche | El Naranjo | *Canis latrans* | 2020/09/29 | -91.1078 | 18.03341 | Cámera trap survey |
| 102 | Mexico | Campeche | Paraiso Nuevo | *Canis latrans* | 2020/09/28 | -91.10999 | 18.06383 | Cámera trap survey |
| 103 | Mexico | Campeche | El Naranjo | *Canis latrans* | 2020/09/27 | -91.12263 | 18.05091 | Cámera trap survey |
| 104 | Mexico | Campeche | Santa Gertrudis | *Canis latrans* | 2020/05/04 | -91.747 | 18.35168 | Cámera trap survey |
| 105 | Mexico | Campeche | Nicte Ha | *Canis latrans* | 2020/03/26 | -91.73421 | 18.34157 | Cámera trap survey |
| 106 | Mexico | Campeche | Nicte Ha | *Canis latrans* | 2020/03/15 | -91.74124 | 18.32239 | Cámera trap survey |
| 107 | Mexico | Campeche | Nicte Ha | *Canis latrans* | 2020/03/15 | -91.72806 | 18.31418 | Cámera trap survey |
| 108 | Mexico | Campeche | Balam Ku | *Canis latrans* | 2019/10/08 | -89.88906 | 18.53951 | Cámera trap survey |
| 109 | Mexico | Campeche | Balam Ku | *Canis latrans* | 2019/10/08 | -89.88906 | 18.53951 | Cámera trap survey |
| 110 | Mexico | Campeche | Términos | *Canis latrans* | 2019/09/29 | -90.88907 | 18.53951 | Cámera trap survey |
| 111 | Mexico | Campeche | Balam Ku | *Canis latrans* | 2019/08/17 | -90.07555 | 18.56562 | Cámera trap survey |
| 112 | Mexico | Campeche | Calakmul | *Canis latrans* | 2019/08/14 | -89.8045 | 18.24758 | Cámera trap survey |
| 113 | Mexico | Campeche | Balam Ku | *Canis latrans* | 2019/08/13 | -90.07555 | 18.56562 | Cámera trap survey |
| 114 | Mexico | Campeche | Balam Ku | *Canis latrans* | 2019/08/13 | -90.07555 | 18.56562 | Cámera trap survey |
| 115 | Mexico | Campeche | Balam Ku | *Canis latrans* | 2019/08/11 | -90.1993 | 18.60754 | Cámera trap survey |
| 116 | Mexico | Campeche | Balam Ku | *Canis latrans* | 2019/06/21 | -90.07555 | 18.56562 | Cámera trap survey |
| 117 | Mexico | Campeche | Champotón | *Canis latrans* | 2019/06/13 | -91.02936 | 19.00669 | Cámera trap survey |
| 118 | Mexico | Campeche | Champotón | *Canis latrans* | 2019/06/10 | -91.02936 | 19.00669 | Cámera trap survey |
| 119 | Mexico | Campeche | Champotón | *Canis latrans* | 2019/06/08 | -91.03422 | 18.9748 | Cámera trap survey |
| 120 | Mexico | Campeche | Champotón | *Canis latrans* | 2019/06/01 | -91.02981 | 19.0143 | Cámera trap survey |
| 121 | Mexico | Campeche | Champotón | *Canis latrans* | 2019/06/01 | -91.02981 | 19.0143 | Cámera trap survey |
| 122 | Mexico | Campeche | Champotón | *Canis latrans* | 2019/05/31 | -91.02981 | 19.0143 | Cámera trap survey |
| 123 | Mexico | Campeche | Champotón | *Canis latrans* | 2019/05/25 | -91.02394 | 19.01172 | Cámera trap survey |
| 124 | Mexico | Campeche | Champotón | *Canis latrans* | 2019/05/15 | -91.02394 | 19.01172 | Cámera trap survey |
| 125 | Mexico | Campeche | Champotón | *Canis latrans* | 2019/05/14 | -91.02394 | 19.01172 | Cámera trap survey |
| 126 | Mexico | Campeche | Champotón | *Canis latrans* | 2019/05/09 | -91.01905 | 19.00178 | Cámera trap survey |
| 127 | Mexico | Campeche | Champotón | *Canis latrans* | 2019/05/09 | -91.01905 | 19.00178 | Cámera trap survey |
| 128 | Mexico | Campeche | Champotón | *Canis latrans* | 2019/05/07 | -91.03422 | 18.9748 | Cámera trap survey |
| 129 | Mexico | Campeche | Nicte Ha | *Canis latrans* | 2019/04/30 | -91.71818 | 18.31949 | Cámera trap survey |
| 130 | Mexico | Campeche | Centenario | *Canis latrans* | 2019/04/26 | -90.13752 | 18.92574 | Cámera trap survey |
| 131 | Mexico | Campeche | Centenario | *Canis latrans* | 2019/04/26 | -90.12535 | 18.9107 | Cámera trap survey |
| 132 | Mexico | Campeche | Champotón | *Canis latrans* | 2019/04/14 | -91.02936 | 19.00669 | Cámera trap survey |
| 133 | Mexico | Campeche | Centenario | *Canis latrans* | 2019/03/31 | -90.1018 | 18.91084 | Cámera trap survey |
| 134 | Mexico | Campeche | Champotón | *Canis latrans* | 2019/03/16 | -91.02936 | 19.00669 | Cámera trap survey |
| 135 | Mexico | Campeche | Champotón | *Canis latrans* | 2019/02/27 | -91.02288 | 18.99514 | Cámera trap survey |
| 136 | Mexico | Campeche | Calakmul | *Canis latrans* | 2019/02/20 | -89.8045 | 18.24758 | Cámera trap survey |
| 137 | Mexico | Campeche | Términos | *Canis latrans* | 2018/10/08 | -90.81954 | 19.17686 | Cámera trap survey |
| 138 | Mexico | Campeche | Balam Ku | *Canis latrans* | 2018/08/26 | -90.06799 | 18.26869 | Cámera trap survey |
| 139 | Mexico | Campeche | Balam Ku | *Canis latrans* | 2018/08/26 | -90.09084 | 18.30372 | Cámera trap survey |
| 140 | Mexico | Campeche | Balam Ku | *Canis latrans* | 2018/08/26 | -90.08136 | 18.28631 | Cámera trap survey |
| 141 | Mexico | Campeche | Balam Ku | *Canis latrans* | 2018/08/26 | -90.09697 | 18.32247 | Cámera trap survey |
| 142 | Mexico | Campeche | Balam Ku | *Canis latrans* | 2018/08/26 | -90.10385 | 18.34002 | Cámera trap survey |
| 143 | Mexico | Campeche | Balam Ku | *Canis latrans* | 2018/07/07 | -90.07387 | 18.1642 | Cámera trap survey |
| 144 | Mexico | Campeche | Balam Ku | *Canis latrans* | 2018/07/07 | -90.07803 | 18.18229 | Cámera trap survey |
| 145 | Mexico | Campeche | Términos | *Canis latrans* | 2017/02/06 | -91.66866 | 18.31316 | Cámera trap survey |
| 146 | Mexico | Campeche | Términos | *Canis latrans* | 2016/10/23 | -91.62266 | 18.10134 | Cámera trap survey |
| 147 | Mexico | Campeche | Términos | *Canis latrans* | 2016/10/23 | -91.96411 | 18.1594 | Cámera trap survey |
| 148 | Mexico | Campeche | Términos | *Canis latrans* | 2016/10/23 | -91.76446 | 18.20798 | Cámera trap survey |
| 149 | Mexico | Campeche | Términos | *Canis latrans* | 2015/10/22 | -91.77631 | 18.06471 | Cámera trap survey |
| 150 | Mexico | Campeche | Términos | *Canis latrans* | 2015/10/22 | -91.85524 | 17.9907 | Cámera trap survey |
| 151 | Mexico | Campeche | Términos | *Canis latrans* | 2015/10/22 | -91.74721 | 17.96725 | Cámera trap survey |
| 152 | Mexico | Campeche | Términos | *Canis latrans* | 2014/10/08 | -91.01643 | 18.98644 | Cámera trap survey |
| 153 | Mexico | Yucatán | Dzilam de Bravo | *Canis latrans* | 2024/04/13 | -88.73046 | 21.39183 | Cámera trap survey |
| 154 | Mexico | Yucatán | Buczotz | *Canis latrans* | 2023/05/30 | -88.50205 | 21.17993 | Cámera trap survey |
| 155 | Mexico | Yucatán | Buczotz | *Canis latrans* | 2023/05/29 | -89.33816 | 21.10105 | Cámera trap survey |
| 156 | Mexico | Yucatán | Buczotz | *Canis latrans* | 2023/05/28 | -89.27007 | 21.13424 | Cámera trap survey |
| 157 | Mexico | Yucatán | Tizimín | *Canis latrans* | 2023/03/09 | -87.59843 | 21.35339 | Cámera trap survey |
| 158 | Mexico | Yucatán | Dzilam de Bravo | *Canis latrans* | 2023/03/02 | -88.72967 | 21.39086 | Cámera trap survey |
| 159 | Mexico | Yucatán | Tizimín | *Canis latrans* | 2023/02/25 | -87.59843 | 21.35339 | Cámera trap survey |
| 160 | Mexico | Yucatán | Tizimín | *Canis latrans* | 2022/07/12 | -87.6087 | 21.33271 | Cámera trap survey |
| 161 | Mexico | Yucatán | Dzilam de Bravo | *Canis latrans* | 2022/05/18 | -88.73903 | 21.39253 | Cámera trap survey |
| 162 | Mexico | Yucatán | Tizimín | *Canis latrans* | 2022/05/14 | -87.61518 | 21.36143 | Cámera trap survey |
| 163* | Mexico* | Yucatán* | Tekax* | *Canis latrans* | 2022/04/26 | -89.45123 | 19.85074 | Cámera trap survey* |
| 164 | Mexico | Yucatán | Tizimín | *Canis latrans* | 2022/03/16 | -87.60634 | 21.34637 | Cámera trap survey |
| 165 | Mexico | Yucatán | Tizimín | *Canis latrans* | 2022/02/10 | -87.61518 | 21.36143 | Cámera trap survey |
| 166 | Mexico | Yucatán | Tizimín | *Canis latrans* | 2022/02/10 | -87.61518 | 21.36143 | Cámera trap survey |
| 167 | Mexico | Yucatán | Tizimín | *Canis latrans* | 2022/02/09 | -87.60801 | 21.36534 | Cámera trap survey |
| 168 | Mexico | Yucatán | Oxkutzcab | *Canis latrans* | 2021/12/28 | -89.54868 | 20.12099 | Cámera trap survey |
| 169 | Mexico | Yucatán | Xbec | *Canis latrans* | 2018/10/06 | -88.7941 | 21.26865 | Cámera trap survey |
| 170 | Mexico | Yucatán | Tizimín | *Canis latrans* | 2015/07/03 | -87.65583 | 21.32397 | Cámera trap survey |
| 171 | Mexico | Yucatán | Tizimín | *Canis latrans* | 2015/07/03 | -87.66152 | 21.32927 | Cámera trap survey |
| 172 | Mexico | Yucatán | Tizimín | *Canis latrans* | 2015/07/03 | -87.66152 | 21.32927 | Cámera trap survey |
| 173 | Mexico | Yucatán | Tizimín | *Canis latrans* | 2015/05/17 | -87.55671 | 21.3596 | Cámera trap survey |
| 174 | Mexico | Yucatán | Tizimín | *Canis latrans* | 2013/05/24 | -87.60948 | 21.33297 | Cámera trap survey |
| 175 | Mexico | Yucatán | Tizimín | *Canis latrans* | 2013/05/24 | -87.59348 | 21.32134 | Cámera trap survey |
| 176 | Mexico | Quintana Roo | Bacalar | *Canis latrans* | 2024/10/09 | -88.1271 | 19.01398 | Cámera trap survey |
| 177 | Guatemala | Petén | Laguna del Tigre NP | *Canis latrans* | 2024/07/09 | -90.79298 | 17.24038 | Cámera trap survey |
| 178 | Guatemala | El Petén | Maya BR | *Canis latrans* | 2024/04/27 | -89.8753 | 17.17496 | Cámera trap survey |
| 179 | Guatemala | Sololá | Santa Lacía Utatlán | *Canis latrans* | 2024/04/18 | -91.25667 | 14.77833 | Cámera trap survey |
| 180 | Guatemala | El Petén | Maya BR | *Canis latrans* | 2024/04/16 | -89.44613 | 17.21085 | Cámera trap survey |
| 181 | Guatemala | El Petén | Maya BR | *Canis latrans* | 2024/02/27 | -89.48695 | 17.17314 | Cámera trap survey |
| 182 | Guatemala | El Petén | Maya BR | *Canis latrans* | 2024/02/27 | -89.47074 | 17.19149 | Cámera trap survey |
| 183 | Guatemala | El Petén | Maya BR | *Canis latrans* | 2024/02/27 | -89.44613 | 17.21085 | Cámera trap survey |
| 184 | Guatemala | Petén | Sierra del Lacandón NP | *Canis latrans* | 2023/06/21 | -90.9493 | 16.91226 | Cámera trap survey |
| 185 | Guatemala | San Marcos | San Pedro | *Canis latrans* | 2023/06/10 | -91.75611 | 14.97083 | Cámera trap survey |
| 186 | Guatemala | Sacatepequez | San Juan Alotenango | *Canis latrans* | 2022/09/05 | -90.82389 | 14.45417 | Cámera trap survey |
| 187 | Guatemala | Baja Verapaz | Purulhá | *Canis latrans* | 2022/03/06 | -90.21916 | 15.21556 | Cámera trap survey |
| 188 | Guatemala | Zacapa | Pueblo Modelo | *Canis latrans* | 2022/02/19 | -89.537 | 14.999 | Cámera trap survey |
| 189 | Guatemala | Zacapa | Usumatlán | *Canis latrans* | 2020/08/10 | -89.85623 | 15.01468 | Cámera trap survey |
| 190 | Guatemala | Zacapa | Usumatlán | *Canis latrans* | 2020/06/05 | -89.85253 | 15.02068 | Cámera trap survey |
| 191 | Guatemala | Zacapa | Usumatlán | *Canis latrans* | 2020/05/07 | -89.85301 | 15.02148 | Cámera trap survey |
| 192 | Guatemala | Zacapa | Usumatlán | *Canis latrans* | 2020/03/18 | -89.85301 | 15.02148 | Cámera trap survey |
| 193 | Guatemala | Zacapa | Usumatlán | *Canis latrans* | 2020/01/24 | -89.86098 | 15.02756 | Cámera trap survey |
| 194 | Guatemala | Zacapa | Usumatlán | *Canis latrans* | 2020/01/06 | -89.85519 | 15.02311 | Cámera trap survey |
| 195 | Guatemala | Zacapa | Usumatlán | *Canis latrans* | 2020/01/06 | -89.85303 | 15.01929 | Cámera trap survey |
| 196 | Guatemala | Zacapa | Usumatlán | *Canis latrans* | 2020/01/06 | -89.85303 | 15.01929 | Cámera trap survey |
| 197 | Guatemala | Zacapa | Usumatlán | *Canis latrans* | 2020/01/06 | -89.85697 | 15.02462 | Cámera trap survey |
| 198 | Guatemala | Zacapa | Usumatlán | *Canis latrans* | 2020/01/06 | -89.86476 | 15.02923 | Cámera trap survey |
| 199 | Guatemala | El Petén | Maya BR | *Canis latrans* | 2019/12/23 | -90.35042 | 17.26055 | Cámera trap survey |
| 200 | Guatemala | El Petén | Maya BR | *Canis latrans* | 2019/12/23 | -90.36235 | 17.21818 | Cámera trap survey |
| 201 | Guatemala | Zacapa | Usumatlán | *Canis latrans* | 2019/12/22 | -89.85519 | 15.02311 | Cámera trap survey |
| 202 | Guatemala | El Petén | Maya BR | *Canis latrans* | 2019/12/04 | -90.32578 | 17.23875 | Cámera trap survey |
| 203 | Guatemala | El Petén | Maya BR | *Canis latrans* | 2019/10/13 | -89.45454 | 17.68927 | Cámera trap survey |
| 204 | Guatemala | El Petén | Maya BR | *Canis latrans* | 2019/10/13 | -89.5121 | 17.711 | Cámera trap survey |
| 205 | Guatemala | El Petén | Maya BR | *Canis latrans* | 2019/10/13 | -89.37157 | 17.73355 | Cámera trap survey |
| 206 | Guatemala | El Petén | Maya BR | *Canis latrans* | 2019/10/13 | -89.37157 | 17.73355 | Cámera trap survey |
| 207 | Guatemala | El Petén | Maya BR | *Canis latrans* | 2019/10/13 | -89.34724 | 17.76312 | Cámera trap survey |
| 208 | Guatemala | El Petén | Maya BR | *Canis latrans* | 2019/10/13 | -89.34724 | 17.76312 | Cámera trap survey |
| 209 | Guatemala | El Petén | Maya BR | *Canis latrans* | 2019/10/13 | -89.30566 | 17.74907 | Cámera trap survey |
| 210 | Guatemala | El Petén | Maya BR | *Canis latrans* | 2019/10/06 | -90.34606 | 17.4996 | Cámera trap survey |
| 211 | Guatemala | El Progreso | San Agustín Acasaguastlán | *Canis latrans* | 2019/04/29 | -90.03313 | 14.90326 | Cámera trap survey |
| 212 | Guatemala | El Progreso | San Agustín Acasaguastlán | *Canis latrans* | 2019/04/29 | -90.03313 | 14.90326 | Cámera trap survey |
| 213 | Guatemala | El Progreso | San Agustín Acasaguastlán | *Canis latrans* | 2019/03/13 | -90.03313 | 14.90326 | Cámera trap survey |
| 214 | Guatemala | El Progreso | San Agustín Acasaguastlán | *Canis latrans* | 2019/03/12 | -90.03313 | 14.90326 | Cámera trap survey |
| 215 | Guatemala | El Progreso | San Agustín Acasaguastlán | *Canis latrans* | 2019/03/09 | -90.03313 | 14.90326 | Cámera trap survey |
| 216 | Guatemala | El Progreso | San Agustín Acasaguastlán | *Canis latrans* | 2019/03/08 | -90.03313 | 14.90326 | Cámera trap survey |
| 217 | Guatemala | El Progreso | San Agustín Acasaguastlán | *Canis latrans* | 2019/03/08 | -90.03313 | 14.90326 | Cámera trap survey |
| 218 | Guatemala | El Progreso | San Agustín Acasaguastlán | *Canis latrans* | 2019/03/07 | -90.03313 | 14.90326 | Cámera trap survey |
| 219 | Guatemala | El Progreso | San Agustín Acasaguastlán | *Canis latrans* | 2019/03/06 | -90.03313 | 14.90326 | Cámera trap survey |
| 220 | Guatemala | El Progreso | San Agustín Acasaguastlán | *Canis latrans* | 2019/03/05 | -90.03313 | 14.90326 | Cámera trap survey |
| 221 | Guatemala | El Progreso | San Agustín Acasaguastlán | *Canis latrans* | 2019/02/19 | -90.03313 | 14.90326 | Cámera trap survey |
| 222 | Guatemala | El Progreso | San Agustín Acasaguastlán | *Canis latrans* | 2019/02/16 | -90.03313 | 14.90326 | Cámera trap survey |
| 223 | Guatemala | El Progreso | San Agustín Acasaguastlán | *Canis latrans* | 2019/02/16 | -90.03313 | 14.90326 | Cámera trap survey |
| 224 | Guatemala | El Progreso | San Agustín Acasaguastlán | *Canis latrans* | 2019/02/11 | -90.03313 | 14.90326 | Cámera trap survey |
| 225 | Guatemala | El Progreso | San Agustín Acasaguastlán | *Canis latrans* | 2019/01/23 | -90.03313 | 14.90326 | Cámera trap survey |
| 226 | Guatemala | El Progreso | San Agustín Acasaguastlán | *Canis latrans* | 2019/01/21 | -90.03313 | 14.90326 | Cámera trap survey |
| 227 | Guatemala | El Progreso | San Agustín Acasaguastlán | *Canis latrans* | 2019/01/17 | -90.03313 | 14.90326 | Cámera trap survey |
| 228 | Guatemala | El Progreso | San Agustín Acasaguastlán | *Canis latrans* | 2019/01/15 | -90.03031 | 14.90283 | Cámera trap survey |
| 229 | Guatemala | El Progreso | San Agustín Acasaguastlán | *Canis latrans* | 2019/01/14 | -90.03031 | 14.90283 | Cámera trap survey |
| 230 | Guatemala | El Petén | Maya BR | *Canis latrans* | 2017/12/01 | -89.78798 | 17.05176 | Cámera trap survey |
| 231 | Guatemala | El Petén | Maya BR | *Canis latrans* | 2017/11/07 | -89.66391 | 17.01242 | Cámera trap survey |
| 232 | Guatemala | El Petén | Maya BR | *Canis latrans* | 2017/10/14 | -89.83332 | 17.09531 | Cámera trap survey |
| 233 | Guatemala | El Petén | Maya BR | *Canis latrans* | 2017/10/14 | -89.54179 | 16.95275 | Cámera trap survey |
| 234 | Guatemala | El Petén | Maya BR | *Canis latrans* | 2017/10/12 | -89.61048 | 17.08206 | Cámera trap survey |
| 235 | Guatemala | El Petén | Maya BR | *Canis latrans* | 2017/10/07 | -89.71412 | 17.03095 | Cámera trap survey |
| 236 | Guatemala | El Petén | Maya BR | *Canis latrans* | 2017/09/25 | -89.62859 | 17.05703 | Cámera trap survey |
| 237 | Guatemala | El Petén | Maya BR | *Canis latrans* | 2017/09/16 | -89.66306 | 17.0538 | Cámera trap survey |
| 238 | Guatemala | El Petén | Maya BR | *Canis latrans* | 2017/09/16 | -89.72539 | 17.04011 | Cámera trap survey |
| 239 | Guatemala | El Petén | Maya BR | *Canis latrans* | 2017/09/03 | -89.57947 | 17.063 | Cámera trap survey |
| 240 | Guatemala | Petén | Sierra del Lacandón NP | *Canis latrans* | 2015/07/26 | -91.14938 | 17.02294 | Cámera trap survey |
| 241 | Guatemala | El Petén | Maya BR | *Canis latrans* | 2013/05/26 | -89.3227 | 17.37569 | Cámera trap survey |
| 242 | Guatemala | El Petén | Maya BR | *Canis latrans* | 2013/05/26 | -89.26772 | 17.42465 | Cámera trap survey |
| 243 | Guatemala | El Petén | Maya BR | *Canis latrans* | 2013/05/26 | -89.23668 | 17.44042 | Cámera trap survey |
| 244 | Belize | Orange Walk | Bergen's Gate | *Canis latrans* | 2024/05/10 | -88.73002 | 17.6645 | Cámera trap survey |
| 245 | Belize | Orange Walk | Bergen's Gate | *Canis latrans* | 2024/07/10 | -88.73942 | 17.6608 | Cámera trap survey |
| 246 | Belize | Orange Walk | Indian Creek | *Canis latrans* | 2023/10/05 | -88.77723 | 17.75957 | Cámera trap survey |
| 247 | Belize | Orange Walk | Hillbank Station | *Canis latrans* | 2020/03/04 | -88.70015 | 17.59852 | Cámera trap survey |
| 248 | Belize | Orange Walk | Yalbac Area | *Canis latrans* | 2019/06/05 | -88.79527 | 17.51297 | Cámera trap survey |
| 249 | Belize | Orange Walk | Yalbac Area | *Canis latrans* | 2019/06/05 | -88.79527 | 17.51297 | Cámera trap survey |
| 250 | Belize | Orange Walk | Yalbac Area | *Canis latrans* | 2019/06/05 | -88.79527 | 17.51297 | Cámera trap survey |
| 251 | Belize | Orange Walk | Yalbac Area | *Canis latrans* | 2019/06/05 | -88.79998 | 17.51293 | Cámera trap survey |
| 252 | Belize | Orange Walk | Sierra de Agua | *Canis latrans* | 2017/07/07 | -88.77923 | 17.57395 | Cámera trap survey |
| 253 | Mexico | Campeche | Champotón | *Canis latrans* | 2022/09/22 | -90.59026 | 19.09911 | iNaturalist/GBIF |
| 254 | Mexico | Quintana Roo | Lázaro Cárdenas | *Canis latrans* | 2021/05/26 | -87.34292 | 20.96845 | iNaturalist/GBIF |
| 255 | Mexico | Quintana Roo | Benito Juárez | *Canis latrans* | 2017/10/24 | -86.8558 | 20.98746 | iNaturalist/GBIF |
| 256 | Mexico | Yucatán | Motúl | *Canis latrans* | 2024/01/21 | -89.23007 | 21.03128 | iNaturalist/GBIF |
| 257 | Mexico | Yucatán | Mérida | *Canis latrans* | 2022/09/12 | -89.60421 | 21.14948 | iNaturalist/GBIF |
| 258 | Mexico | Yucatán | Progreso | *Canis latrans* | 2022/02/02 | -89.80345 | 21.15556 | iNaturalist/GBIF |
| 259 | Mexico | Yucatán | Peto | *Canis latrans* | 2021/07/04 | -88.8561 | 19.91662 | iNaturalist/GBIF |
| 260 | Mexico | Yucatán | Dzemul | *Canis latrans* | 2020/08/19 | -89.36667 | 21.25145 | iNaturalist/GBIF |
| 261 | Mexico | Yucatán | Mérida | *Canis latrans* | 2019/08/12 | -89.61905 | 21.15582 | iNaturalist/GBIF |
| 262 | Mexico | Yucatán | Ucú | *Canis latrans* | 2018/11/09 | -89.80846 | 21.15525 | iNaturalist/GBIF |
| 263 | Mexico | Yucatán | Ixil | *Canis latrans* | 2018/10/20 | -89.45386 | 21.26308 | iNaturalist/GBIF |
| 264 | Mexico | Yucatán | Progreso | *Canis latrans* | 2015/12/12 | -89.82858 | 21.22019 | iNaturalist/GBIF |
| 265 | Mexico | Yucatán | Celestún | *Canis latrans* | 2010/05/09 | -90.33958 | 20.88133 | iNaturalist/GBIF |
| 266 | Guatemala | Guatemala | San José Pinula | *Canis latrans* | 2024/04/27 | -90.41016 | 14.54478 | iNaturalist/GBIF |
| 267 | Guatemala | Izabal | Juyamá | *Canis latrans* | 2023/11/22 | -88.91495 | 15.2983 | iNaturalist/GBIF |
| 268 | Guatemala | Escuintla | Santa Lucia | *Canis latrans* | 2023/10/26 | -91.06662 | 14.32161 | iNaturalist/GBIF |
| 269 | Guatemala | Huehuetenango | Todos Santos Park | *Canis latrans* | 2023/08/12 | -91.5693 | 15.53017 | iNaturalist/GBIF |
| 270 | Guatemala | Petén | San Francisco | *Canis latrans* | 2023/05/22 | -89.9407 | 16.84315 | iNaturalist/GBIF |
| 271 | Guatemala | Guatemala | Antigua | *Canis latrans* | 2022/02/04 | -90.69198 | 14.5268 | iNaturalist/GBIF |
| 272 | Guatemala | Guatemala | Villa Canales | *Canis latrans* | 2021/05/25 | -90.5562 | 14.35548 | iNaturalist/GBIF |
| 273 | Guatemala | Huehuetenango | Todos Santos Park | *Canis latrans* | 2017/03/29 | -91.55106 | 15.50915 | iNaturalist/GBIF |
| 274 | Belize | Belize | Belize | *Canis latrans* | 2023/09/08 | -88.49265 | 17.31504 | iNaturalist/GBIF |
| 275 | Belize | Cayo | Cayo | *Canis latrans* | 2020/02/25 | -89.07984 | 17.35864 | iNaturalist/GBIF |
| 276 | Belize | Orange Walk | Orange Walk | *Canis latrans* | 2019/02/03 | -89.0171 | 17.65852 | iNaturalist/GBIF |
| 277 | Belize | Cayo | Yalbac Soth Block | *Canis latrans* | 2016/10/21 | -88.98552 | 17.38997 | iNaturalist/GBIF |
| 278 | Belize | Cayo | Yalbac Soth Block | *Canis latrans* | 2015/09/15 | -88.95525 | 17.40082 | iNaturalist/GBIF |
| The superscript asterisk (*) indicates a record (ID163) obtained through the project named “Acciones de implementación de la Estrategia Estatal REDD+ (EEREDD+) del Estado de Yucatán para la inversión público privada en el desarrollo rural sustentable: emprendimiento sustentable en milpa maya, apicultura, huertos de traspatio y PSA Bioculturales e innovadores”, in its biodiversity component, under the coordination of the Secretariat of Sustainable Development (SDS) of Yucatan; the Intermunicipal Biocultural Board of Puuc (JIBIOPUUC) and the World Wide Fund for Nature (WWF), with funding from the GCF Task Force. (JIBIOPUUC) y El Fondo Mundial para la Naturaleza (WWF), con financiamiento del GCF Task Force. | | | | | | | | |
