# Supplementary material for: Coyote Range Expansion in the Human‐Modified Tropics of Mesoamerica
Source: Ecol Evol. 2026 Mar 2;16(3):e73184. doi: 10.1002/ece3.73184 (PMC12953002; doi:10.1002/ece3.73184)
Supplement: Supplementary file 2 — Figures S1–S2: ece373184‐sup‐0002‐FigureS1‐S2.docx. [file ECE3-16-e73184-s002.docx]

**Supplementary material**


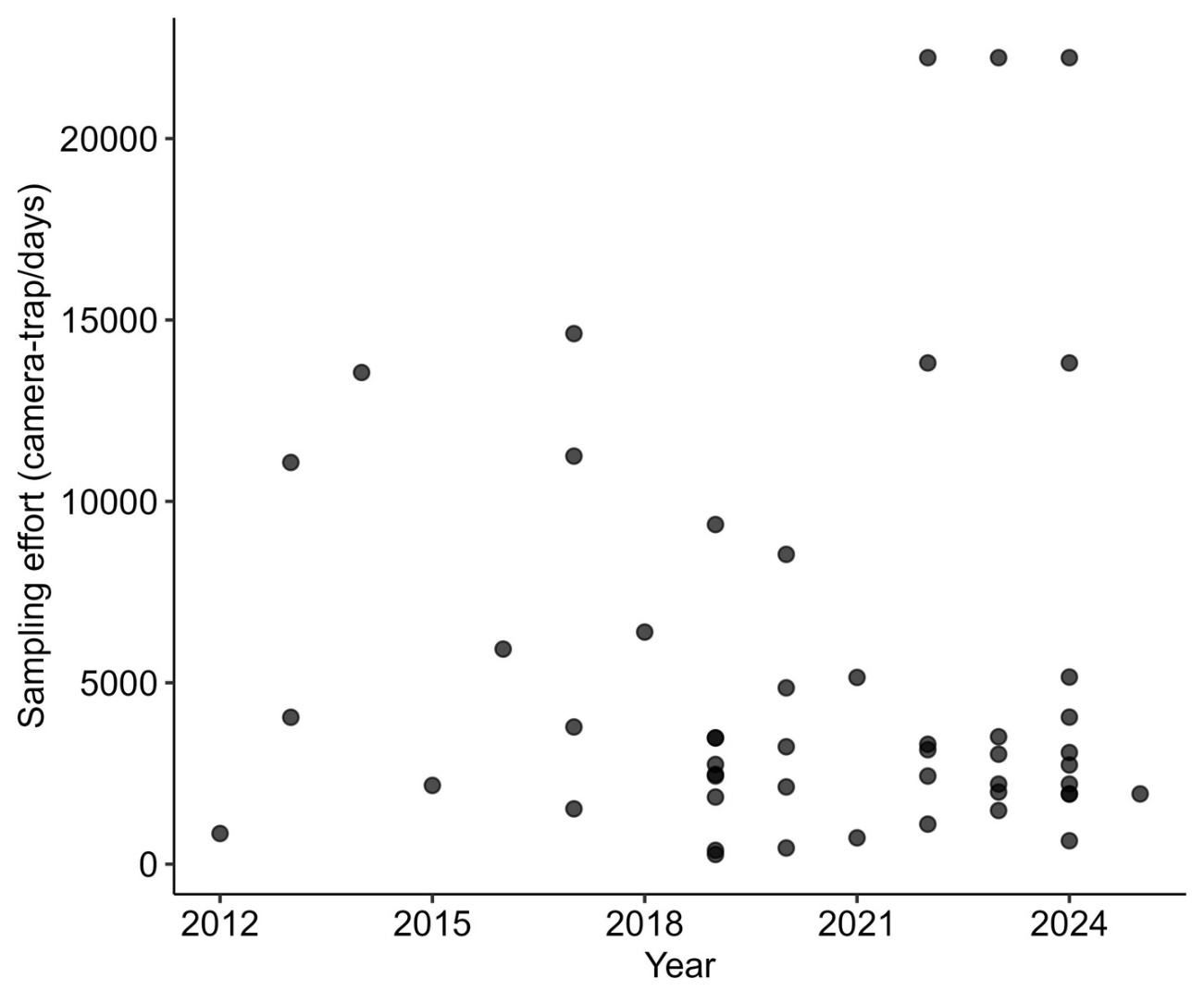


**Figure S1.** Annual variation in camera-trap sampling effort across the study region between 2012 and 2025. Points represent the total sampling effort (camera-trap days) for each independent monitoring project conducted in a given year.


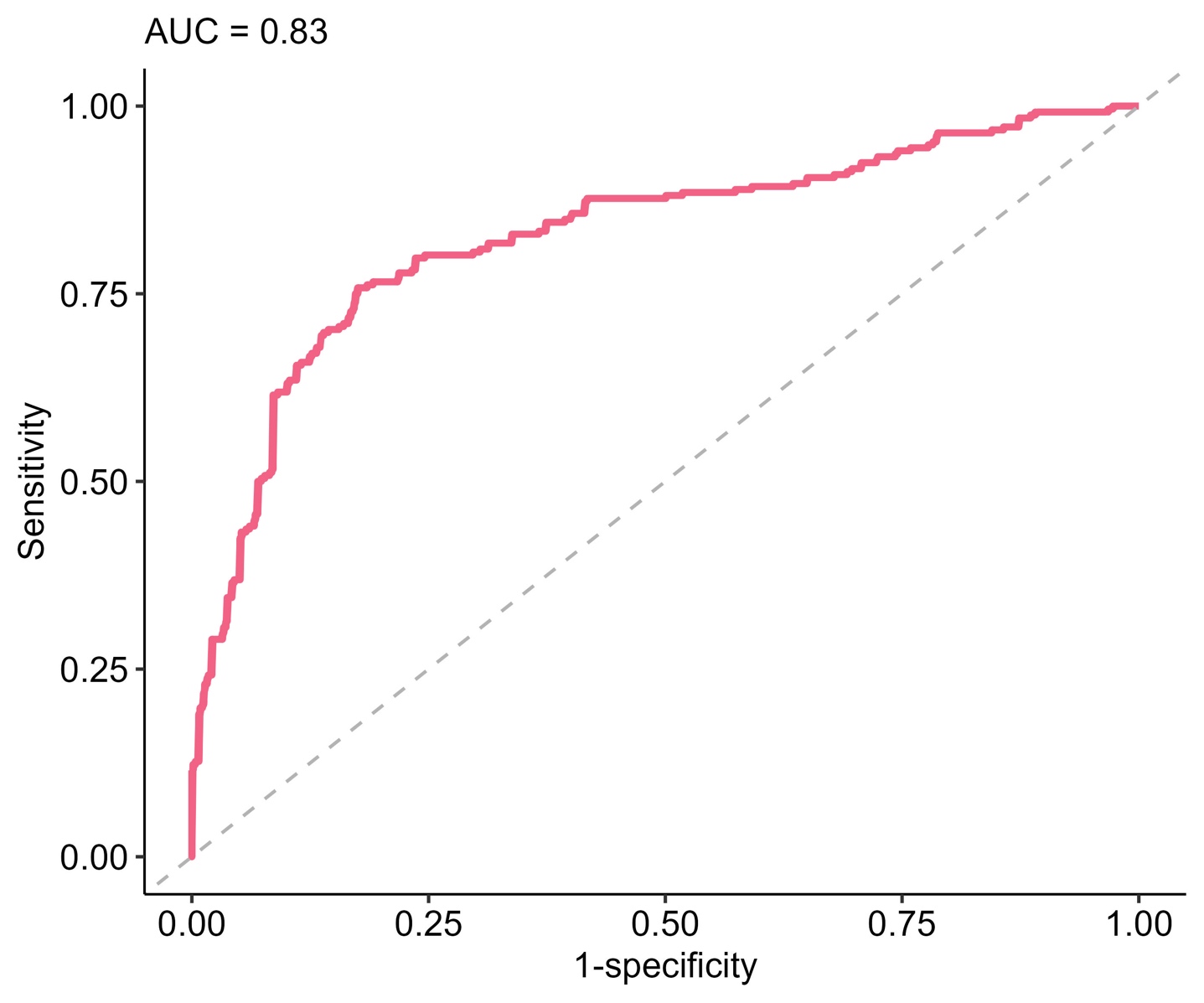


**Figure S2.** Receiver Operating Characteristic (ROC) curve showing the discriminative performance of the generalized linear model used to predict coyote (Canis latrans) occurrence. The curve illustrates the relationship between sensitivity and 1–specificity across probability thresholds. The dashed line represents the null expectation of random classification. Model performance was high (AUC = 0.83; 95% CI: 0.80–0.86), indicating excellent discrimination between coyote presence and absence locations.
